# Enhancing protein immunogenicity prediction via uncertainty weighted deep ensemble

**DOI:** 10.1093/oxfimm/iqag008

**Published:** 2026-03-27

**Authors:** Alif Bin Abdul Qayyum, Amir Hossein Rahmati, Xiaoning Qian, Byung-Jun Yoon

**Affiliations:** Department of Electrical and Computer Engineering, Texas A&M University, College Station, TX, 77843, United States; Department of Electrical and Computer Engineering, Texas A&M University, College Station, TX, 77843, United States; Department of Electrical and Computer Engineering, Texas A&M University, College Station, TX, 77843, United States; Department of Computer Science and Engineering, Texas A&M University, College Station, TX, 77843, United States; Computing and Data Sciences, Brookhaven National Laboratory, Upton, NY, 11973, United States; Department of Electrical and Computer Engineering, Texas A&M University, College Station, TX, 77843, United States; Computing and Data Sciences, Brookhaven National Laboratory, Upton, NY, 11973, United States

**Keywords:** Deep Ensemble, Uncertainty Quantification (UQ), Uncertainty-aware Prediction, Protein Language Model (PLM), Immunogenicity Prediction, Vaccine Design

## Abstract

Recent advances in machine learning (ML) have significantly improved data-driven protein immunogenicity prediction. Although these ML models perform well on natural proteins, their practical utility is limited by the absence of reliable uncertainty estimates for their predictions. The objective of this work is to develop a method that incorporates predictive uncertainty to achieve uncertainty-aware protein immunogenicity prediction. We introduce *DUNE* (*D*eep *UN*certainty-weighted *E*nsemble), a novel method designed to integrate predictive uncertainty into ML models. DUNE is built to enhance prediction performance by incorporating uncertainty estimates from probabilistic member models in an ensemble into the final prediction. Experimental results demonstrate that incorporating uncertainty estimates through DUNE significantly enhances protein immunogenicity predictive performance. Our proposed DUNE method outperforms existing deterministic single learners and various other deterministic and probabilistic ensemble-based classification strategies. DUNE provides a more reliable and robust framework for protein immunogenicity prediction. By achieving uncertainty-aware prediction, DUNE can improve the trustworthiness and practical utility of ML models in therapeutic antigen design.

## Introduction

Evaluating and monitoring protein properties are of significant importance, leading to substantial efforts in accurately and efficiently predicting safety-related protein properties, e.g. immunogenicity [1–3]. Recent advances in computational power and modeling has enabled novel protein designs [4, 5]. Alongside, the advent of ML based protein structure prediction models, e.g. AlphaFold (AF), ESMFold, and protein language models (PLMs) [6–10] has inspired many scientists and engineers to use hidden representations of them to model relationships between proteins and their properties, since hidden representations are believed as rich enough features to capture the correlations. These developments have paved the way for a coordinated effort to model the relationship between protein properties and their corresponding sequence-structural features, extracted from protein structure prediction models and protein language models [11].

Machine learning (ML) models designed to predict protein properties are typically developed using a rigorous train/validation/test dataset split. This methodology involves training the model on the training set, subsequently tuning hyper-parameters and performing initial performance assessments on the validation set, and finally evaluating generalization performance and robustness on a held-out test set. Despite these stringent evaluation protocols, concerns persist regarding the composition of these datasets, as highlighted by recent research [12]. A critical limitation, furthermore, is the general absence of uncertainty estimates from these predictors, which are indispensable for statistically sound decision-making in protein design and characterization.

Uncertainty quantification (UQ) is an active research area of studying how to quantify uncertainties in models, not limited to statistical models [13, 14], including physics-based models [15–17]. As models have grown larger and more nonlinear, specifically deep learning models, applications of UQ methods for classical statistical modeling [18–20] are computationally expensive and inaccurate.

Ensemble based prediction with ML models has shown better performance than single learners [21–25]. Further integration of uncertainty estimates into ensemble approaches can enable uncertainty-aware prediction for deep ensemble models. Also, most of the existing UQ metrics do not explicitly take into consideration the variations of predictions from probabilistic prediction models, necessitating a novel UQ metric specifically designed for probabilistic prediction models.

In this work, we explore these two aforementioned aspects:

- **DUNE:** We propose a novel methodology for protein property prediction, **D**eep **UN**certainty-weighted **E**nsemble, which integrates uncertainty estimates into deep ensemble models for enhanced protein property prediction.
- **CDiv:** We propose, **C**ross **Div**ergence, a novel uncertainty quantification metric specifically designed for probabilistic models that explicitly takes into account the variations in predictions, unlike many of the existing UQ metrics.

We showcase how incorporating uncertainty estimates can enhance immunogenicity predictive performance. For an extensive evaluation of our proposed approach, we also evaluate our models in another safety and efficacy related protein property—toxicity. We also report investigative evaluations on the effectiveness of integration of uncertainty estimates into ensemble based approach, alongside a case study for investigating the effectiveness of our proposed DUNE method in identifying clinically validated vaccine targets.

## Related Works

**Protein Property Prediction:** The growing fields of protein engineering and therapeutic development demand precise and efficient ways to characterize crucial protein properties, especially those affecting safety and efficacy. The ability of a protein to elicit immune response (immunogenicity) is a critical concern in the development of biopharmaceuticals, vaccines, and gene therapies. To address this, VenusVaccine offers a deep learning solution that uses a dual attention mechanism to combine pretrained latent vector representations of protein sequences and structures [11].

Equally important is predicting protein toxicity, particularly for proteins used in therapeutic or industrial applications. Proteins can be toxic through various means, including direct cell damage, disrupting physiological processes, or accumulating to harmful levels. ToxDL 2.0 tackles this with a new multimodal deep learning model that integrates evolutionary and structural information from a pre-trained language model and AlphaFold2 [26].

Furthermore, with more new proteins appearing in food, pharmaceuticals, and industrial products, assessing their potential to cause allergies is vital for public health. AllergenAI provides a new AI-based tool to quantify this allergenic potential based solely on protein sequences, setting it apart from previous tools that also used physicochemical properties and sequence homology [27].

While machine learning has revolutionized safety and efficacy related protein property prediction, there is a notable gap in research concerning the application of UQ in this field. Integrating UQ into existing data-driven protein property prediction methods requires new research to boost their predictive performance.

**Deep Ensemble Meets UQ:** To improve classification performance with deep learning models, there has been a trend toward using ensemble methods, which allow individual members to specialize in predictions for sparser data regions. Uncertainty Voting (UVote) is a recently proposed ensemble approach designed to tackle imbalanced regression problems [28]. It integrates recent advancements in probabilistic deep learning, and its core mechanism involves deriving the final prediction from the least uncertain member of the deep learning model ensemble.

The integration of UQ into deep ensemble methods for safety and efficacy related protein property prediction, however, remains largely unexplored, highlighting a significant need for novel research endeavors.

## Preliminary

### Uncertainty Quantification

All models and data are inherently imperfect. This imperfection primarily stems from two sources: the underlying assumptions made during model derivation and measurement errors present in the data collection process. Accurately assessing these uncertainties can significantly enhance the reliability of model predictions. UQ aims to estimate the confidence in a Deep Neural Network (DNN) prediction, going beyond just its accuracy. However, quantifying these uncertainties is often non-trivial. UQ methods are typically problem-specific and can be computationally expensive to implement.

The most commonly utilized way to address model uncertainty is through a Bayesian neural network (BNN) [29]. A BNN accounts for parameter uncertainty by placing a prior distribution over its model parameters. The goal is then to infer the posterior distribution of these parameters, which provides a theoretical basis for understanding the model’s inherent uncertainty.

To address the high computational and memory demands of Bayesian Neural Networks (BNNs), various approximation methods have been developed. In this work, we focus on five representative methods, namely MCD [30], DKL [31], Laplace approximation [32], SWAG [33], and VBLL [34], which we briefly summarize in the following:

**Monte-Carlo Dropout (MCD)** estimates uncertainty by interpreting stochastic forward passes as approximate Bayesian inference in deep Gaussian processes.

**Stochastic Variational Deep Kernel Learning (DKL)** combines deep networks with Gaussian processes using stochastic variational inference for scalable, flexible uncertainty modeling.

**Laplace Approximation (LA)** fits a Gaussian around the MAP estimate using the Hessian of the log-posterior to model uncertainty efficiently.

**Stochastic Weight Averaging Gaussian (SWAG)** uses SGD trajectories to approximate a Gaussian posterior over weights, enabling Bayesian model averaging with low overhead.

**Variational Bayesian Last Layers (VBLL)** maintains a posterior only over the last layer via a deterministic variational approach, yielding fast, sampling-free uncertainty estimates.

### Deep Ensemble

A deep ensemble model leverages the *wisdom of crowds* by combining the predictions of multiple individual deep neural networks (DNNs) [21, 35]. Instead of relying on a single and potentially overconfident model, a deep ensemble trains several distinct DNNs, often with different random initializations, data subsets, or even architectures. Deep ensemble can achieve greater predictive accuracy, improved robustness, and more reliable uncertainty estimates by averaging or combining the outputs of these diverse member models than any single component model [36, 37]. This is because different models within the ensemble may capture distinct aspects of the data and make uncorrelated errors, leading to a more robust consensus. Deep ensembles are particularly effective for tasks requiring uncertainty quantification, as the variability in predictions across the ensemble members can provide a measure of confidence [38].

Predictions for classification tasks from any deep ensemble model can be obtained through multiple different approaches. For the sake of comparative evaluation with existing deep ensembles, we choose four existing approaches as baselines. Brief descriptions of those are as follows:

- **Majority Voting (MVote)**: This refers to the scenario where we take the verdict of the majority among members in an ensemble of deterministic classifier models [39]. An odd number of members is usually selected to constitute the ensemble to avoid tie-breaking.
- **Soft Voting (SVote)**: In another variant of voting based approaches, we take the verdicts of all members on class probabilities and take the average of them, more generally termed as *Soft Voting*, and also known as *Uniform Weighting*.
- **Performance Weighting (PWeight)**: This strategy refers to weighted averaging of predictions from models of an ensemble based on the performance of the models on a held-out dataset, e.g. validation set [40]. This is typically a two-step optimization process: the parameters of the ensemble members are first optimized using the train set, and then member-specific weights are optimized based on each member’s performance on a held-out validation dataset.
- **Uncertainty Voting (UVote)**: Model uncertainty is generally not accounted for in standard majority voting, soft voting or performance weighted schemes. *Uncertainty Voting* is a strategy where an ensemble’s final decision is determined by the least uncertain member [28]. Typically, a Bayesian Neural Network (BNN) variant is used to estimate each model’s uncertainty. The simplest way to identify the least uncertain model is by finding the one with the smallest variation in its probabilistic predictions.

### Protein Language Model

The application of large language models (LLMs)—originally transformative in natural language processing (NLP)—to protein sequences has led to the development of sophisticated protein language models (PLMs) [7, 41, 42]. This modeling approach treats amino acids as analogous to words and full protein sequences as sentences, enabling the use of language modeling techniques in a biological context [43, 44]. Typically trained in a self-supervised manner on large-scale amino acid datasets, these PLMs learn rich contextual representations of residues [7, 42]. As a result, they can function as general-purpose feature extractors for various protein analysis tasks, such as protein fold classification, binding site identification, sub-cellular localization, property and structure prediction.

In this work we take advantage of five most recent state-of-the-art (SOTA) PLMs to acquire protein encodings for use in VenusVaccine [11], the backbone in our experiments. Particularly we use ProstT5 [45], Ankh [46], ESM-2 [9], ProtTrans [47], and ESM-Cambrian [48].

## Method

**Figure 1:**
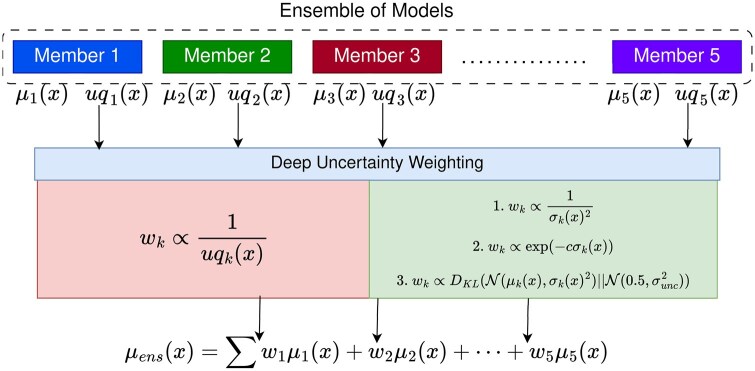
Deep **UN**certainty weighted **e**nsemble. We adopt an ensemble of five probabilistic classifiers and integrate uncertainty estimates into the final protein property classification task. We also propose three different weighting approaches to integrate the uncertainty estimates.

We propose a deep ensemble classifier model that consists of several probabilistic binary classifier models, $M_{k}$. The distinctiveness among these ensemble members lies in their representation of the input protein data. Specifically, each protein is characterized by hidden embeddings generated from a protein language model (PLM). To introduce diversity within the deep ensemble, we have employed five different PLMs, mentioned in the above **Protein Language Model** subsection, for each of the five ensemble members for its protein representations. This approach ensures that the individual models within the ensemble are exposed to different features of the proteins, potentially leading to a more robust and comprehensive collective prediction. In this study, we adopt **VenusVaccine** [11] model architecture for the backbone of each member of the ensemble. Adoption of a probabilistic variant of this architecture is discussed in the **Ensemble Member Architecture** subsection in our **Experiments** section. Figure 1 summarizes the proposed DUNE method.

### DUNE

Each member $M_{k}$ in the deep ensemble provides a predicted probability, $\mu_{k}(x)$, over positive class for protein data *x*, and an estimated uncertainty $UQ_{k}(x)$ over its prediction. To obtain the final prediction from the deep ensemble, we compute the weighted average of the individual predictions from each member within that ensemble.


(1) $$\mu_{\mathrm{ens}}(x)=\sum w_{k}(x)\mu_{k}(x)$$

The weights for each member in that ensemble are inversely proportional to the uncertainty, $UQ_{k}(x)$, of the probabilistic prediction of that corresponding member:


(2) $$w_{k}\propto \frac{1}{UQ_{k}(x)}$$

For the sake of simplicity, we assume each member’s prediction follow a Gaussian distribution, indicated by $P_{M_{k}}(x)=N(\mu_{k}(x),\sigma_{k}{\left(x\right)}^{2})$, where $\sigma_{k}(x)$ indicates the estimated uncertainty of the member’s prediction. We propose three different weighting schemes to determine the values of $w_{k}$ as detailed below:

**(1) Unbiased Weighting (Unbiased):** The weights, $w_{k}(x)$, can be set inversely proportional to the variance of predicted distribution:


(3) $$w_{k}(x)\propto \frac{1}{\sigma_{k}^{2}(x)}.$$

The predicted variance, $\sigma_{\mathrm{ens}}^{2}$ of the ensemble model is $\sum w_{k}^{2}\sigma_{k}^{2}$. Setting the weights $w_{k}$ inversely proportional to corresponding member predicted variances makes $\sigma_{\mathrm{ens}}^{2}$ a constant, resulting $\mu_{\mathrm{ens}}$ being unbiased to any member’s variations in predictions.

**(2) Negative Softmax Weighting (NS):** The weights, $w_{k}(x)$, are assigned proportionally to the negative softmax of the standard deviation of the predicted distribution:


(4) $$w_{k}(x)\propto \;\text{exp}(-c\sigma_{k}(x)),$$

The parameter *c* acts as a control parameter, modulating the influence of the member-specific weights, $w_{k}$. A higher value of *c* amplifies the contribution of members with greater predictive certainty, effectively giving more *mass* to their predictions within the ensemble. This mechanism allows the ensemble to prioritize models that are more confident in their predictions.

**Figure 2:**
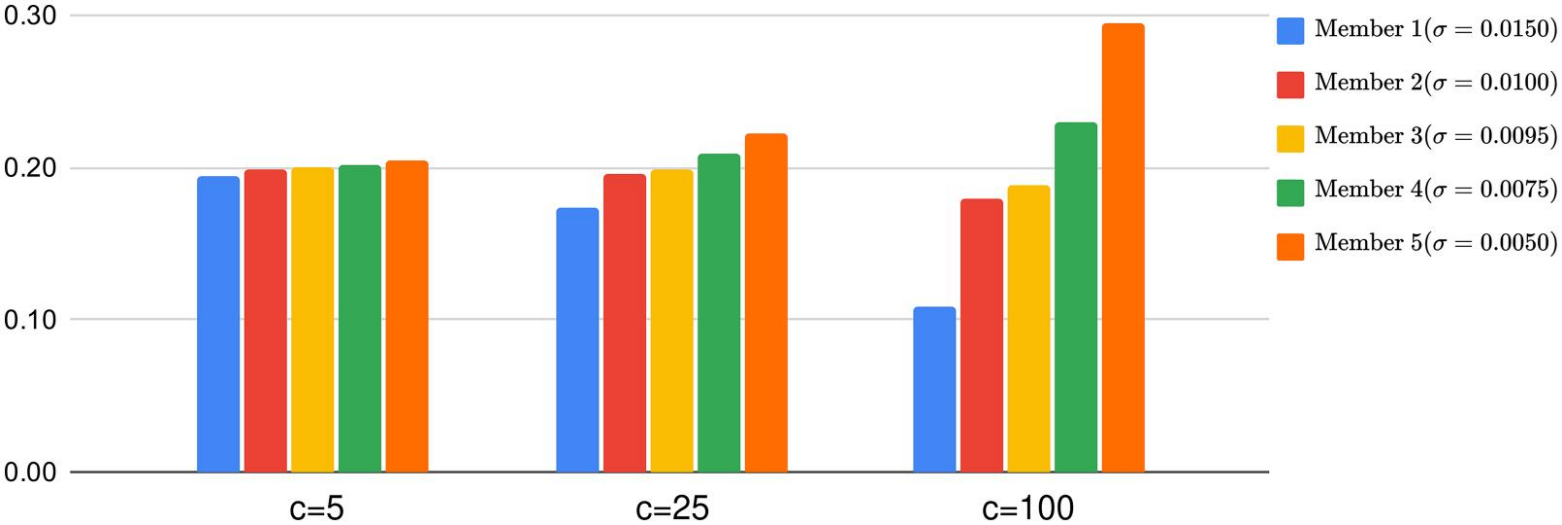
Negative softmax weighting. Members in the ensemble with their corresponding $\sigma_{k}$’s (sorted from highest to lowest) are displayed and the y−axis shows the weights $w_{k}$ for different values of *c*.

Figure 2 summarizes the effect of *c* on weights for corresponding members in the ensemble. An ensemble consisting of 5 members with estimated uncertainties 0.0150, 0.0100, 0.0095, 0.0075 and 0.0050 accordingly and their corresponding weights are displayed. Figure 2 shows that the higher the *c*, the greater portion of the total weights the least uncertain member (with estimated uncertainty, 0.0050, in this example) receives.

Effectively c=∞ evolves into **Uncertainty Voting** method, and c=0 evolves into **Soft Voting**. Negative softmax weighting balances between these two extremes with different values of *c* in the range of (0,∞).

**(3) KL-Divergence Weighting (KLD):** Another weighting scheme is defined as follows:


(5) $$w_{k}\propto D_{KL}(P_{M_{k}}(x)||P_{\mathrm{unc}}).$$

Here the weights, $w_{k}(x)$, are assigned proportionally to the KL-divergence [49, 50] between, $P_{M_{k}}(x)$, the predicted distribution, and $P_{\mathrm{unc}}$, a reference distribution. We use $P_{\mathrm{unc}}=N(0.5,\sigma_{\mathrm{unc}}^{2})$ as the reference distribution. This reference distribution is chosen based on the intuition that, in a classification setting, a model lacking knowledge about an input should assign approximately equal probability to each class. However, in rare cases where the input is inherently ambiguous, the model may confidently predict a balanced probability (e.g. 0.5 in binary classification), which should be reflected in a *low predictive variance*. In contrast, when the model is uncertain due to lack of evidence, it is expected not only to assign equal probabilities, but also to exhibit *high variance* in its prediction. Accordingly, the variance $\sigma_{\text{unc}}^{2}$ in the reference distribution $N(0.5,\sigma_{\text{unc}}^{2})$ controls the sensitivity of the proposed metric to uncertainty. In our experiments, we set $\sigma_{\text{unc}}=0.1$.

An uncertain binary classification model would behave in a way that its prediction would follow a Normal distribution $N(0.5,\sigma_{\text{unc}}^{2})$ with a sufficiently high value for $\sigma_{\text{unc}}$. The reason for selecting $\sigma_{\text{unc}}=0.1$ is the fact that under the probability distribution $N(z;\mu =0.5,\sigma^{2}=0.1^{2})$, the probability P(0.0≤z≤1.0) is almost equal to 1. For a binary classification problem, any value for predicted positive class probability outside the range of [0,1] does not make any sense. Also, a $\sigma_{\text{unc}}$ less than 0.1 would make the model less uncertain. That’s why we model the predicted positive class probability from an uncertain model as ${\hat{y}}_{\text{unc}}∼N(0.5,0.1^{2})$. The objective of estimating predictive uncertainty of a trained model is to identify how differently the trained model behaves from an uncertain model.

To further demonstrate this, we consider the perspective of *evidence theory*, particularly in *evidential deep learning* [51], where a similar intuition emerges. Assuming a symmetric Beta prior for binary classification with parameters $\left(a_{1}=a_{2}=a\right)$, the variance of the resulting Beta distribution becomes: $\text{Var}=\frac{a^{2}}{{\left(2a\right)}^{2}(2a+1)}=\frac{1}{4(2a+1)}$. This gives us a principled way to calibrate the degree of uncertainty: the smaller the *a*, the higher the variance, and thus the more “uncertain” the belief. This can then be mapped to a Gaussian approximation with a corresponding standard deviation and use it to define a reference distribution: $N(0.5,\sigma_{\text{unc}})$.

So whether from a probabilistic (Gaussian) or evidential (Beta) viewpoint, the key idea is to define a *canonical uncertainty distribution* that is centered at 0.5 and sufficiently **broad** to represent maximal ignorance, and then use this distribution as a basis for quantifying how certain or uncertain our model predictions are.

### Cross Divergence

We model the prediction probability from an uncertain probabilistic binary classifier model following a Gaussian distribution $P_{\mathrm{unc}}=N(0.5,\sigma_{\mathrm{unc}}^{2})$ for any binary classification problem.

We evaluate the quantified uncertainty of each member in our deep ensemble model using the following equation for **CDiv** (**C**ross-**Div**ergence):


(6) $$\begin{array}{cc} cd_{k}(x)=\left(y(x)\;\text{log}\;\left(2\mu_{k}(x)\right)+(1-y(x))\;\text{log}\;\left(2-2\mu_{k}(x)\right)\right) & \\ D_{KL}(N(\mu_{k}(x),\sigma_{k}{\left(x\right)}^{2})||N(0.5,\sigma_{\mathrm{unc}}^{2})) & \end{array}$$

Here y(x) denotes the true label for datapoint *x*.

KL-divergence $D_{KL}(P_{M_{k}}||P_{\mathrm{unc}})$ quantifies the dissimilarity between two distributions $P_{M_{k}}\;\text{and}\;P_{\mathrm{unc}}$, with low values indicating high similarity between the two distributions and vice versa [49, 50]. The multiplicative term, $y(x)\;\text{log}\;\left(2\mu_{k}(x)\right)+(1-y(x))\;\text{log}\;\left(2-2\mu_{k}(x)\right)$, gives a positive value if $\mu_{k}(x)$ is at the correct side of the decision boundary, 0.5, whereas it gives a high negative value if $\mu_{k}(x)$ is at the incorrect side of the decision boundary. As a result, the cross-divergence term produces a high positive value if the predicted distribution has a mean close to the true label with a low variance, whereas a very high negative value if the predicted distribution has a mean close to the incorrect label with a low variance, indicating high value inferring low uncertainty and vice versa.

The intuition behind the new Cross-Divergence metric comes from the fact that probabilistic models with high confidence should predict the distribution, $P_{M_{k}}(x)=N(\mu_{k}(x),\sigma_{k}{\left(x\right)}^{2})$, with a mean $\mu_{k}(x)$ close to the true label y(x) and with low variance. To quantify this, we evaluate the distance between predicted distribution and the distribution an uncertain model would provide. However, a model can predict incorrectly and with extreme confidence (mean close to the incorrect label and with a low variance). That’s why we multiply the KL-divergence term with a multiplicative term that produces a positive value for correct predictions and a high negative value for incorrect prediction. Existing UQ metrics do not explicitly take into account the variations in predictions, which is an indication of the uncertainty of the model in its predictions. A highly certain probabilistic model is supposed to show low variations in its predictions. For example, most of the existing UQ metrics only take into account the predicted μ’s, but not the predicted σ’s. So if we have a probabilistic model that gives us the distribution $N(0.8,0.01^{2})$, we would intuitively consider that this is more confident than a model which gives distribution of $N(0.8,0.05^{2})$. However, most of existing UQ metrics would identify both models as equally confident.

To evaluate the quantified uncertainty of an ensemble with *M* number of members, we take the average of the quantified uncertainty of each member of our ensemble:


(7) $$cd(x)=\frac{1}{M}\sum_{k=1}^{M}cd_{k}(x)$$

## Experiments

### Ensemble Member Architecture

We followed **VenusVaccine** [11] model architecture, a Dual Attention mechanism based deep learning model, as the backbone architecture for each member of the ensemble. The VenusVaccine model architecture uses a multi-modal approach for input protein data representation. The three modalities are:

(i) *Sequence Embedding:* We used pre-trained protein language model (PLM) to extract embeddings that represent protein sequences. For example, a protein sequence of length *L* processed by a pre-trained PLM yields an L×V representation, where each Amino Acid is represented by a *V*-dimensional vector. In our proposed ensemble model, each member uses a different PLM to extract sequence embedding.


(8) $$E_{\text{seq}}=PLM_{\text{emb}}(x_{\mathrm{seq}})$$

(ii) *Structural Embedding:* We use ESM3 [10] and FoldSeek [52] model to extract structural features from protein structures predicted by ESMFold [9].


(9) $$x_{\text{structure}}=\mathrm{ESMFold}(x_{\text{seq}})$$

(10) $$E_{\text{esm}3}=\mathrm{ESM}3_{\text{emb}}(x_{\text{structure}})$$

(11) $$x_{\text{esm}3}=\mathrm{CrossAttentio}n_{\theta }^{\text{esm}3}(E_{\text{seq}},E_{\text{esm}3})$$

(12) $$E_{\text{foldseek}}=\mathrm{FoldSee}k_{\text{emb}}(x_{\text{structure}})$$

(13) $$x_{\text{foldseek}}=\mathrm{CrossAttentio}n_{\theta }^{\text{foldseek}}(E_{\text{seq}},E_{\text{foldseek}})$$

(iii) *Physicochemical Descriptors:* Five hand-crafted E-descriptors [53] and three Z-descriptors [54] offer an Amino Acid-level summary of key physicochemical properties, including hydrophobicity and secondary structure propensity.


(14) $$E_{\text{ez}}=EZ_{\text{descriptor}}(x_{\text{seq}})$$

(15) $$x_{ez}=MLP_{\theta }^{ez}(\mathrm{CONCAT}(E_{\text{seq}},E_{\text{ez}}))$$

These features are then concatenated and passed though a MLP network to predict the positive class probability $\hat{y}$ of the corresponding protein.


(16) $$\hat{y}=f_{\theta }(\mathrm{CONCAT}(E_{\text{seq}},x_{\text{esm}3},x_{\text{foldseek}},x_{ez})$$

For probabilistic variant of this architecture, we treat parameters of $f_{\theta }$ as a random variable to enable uncertainty estimation.

### Datasets

We evaluate our models across two protein property datasets: (i) Immunogenicity and (ii) Toxicity.

**Immunogenicity:** We utilized ImmunoDB [11], a comprehensive immunogenicity database containing 7,216 labeled antigens from bacterial, viral, and human sources. Each antigen is categorized as either immunogenic (positive) or non-immunogenic (negative). ImmunoDB was compiled through a meticulous process involving literature curation, database mining, and bioinformatics filtering. The majority of positive samples were sourced from previously published studies. To maintain high data quality, redundant sequences and samples from ambiguous regions were removed. This rigorous curation yielded three distinct subsets: Immuno-Virus, Immuno-Bacteria, and Immuno-Tumor.

In total, this dataset provides **913**/**1562** positive/negative instances in Immuno-Bacteria, **2078**/**1886** in Immuno-Virus, and **300**/**477** in Immuno-Tumor. Table 1 shows the train/validation/test split of the ImmunoDB dataset. For the sake of simplicity, we will interchangeably use Immuno-Virus and Virus for the rest of this paper (similar policy for Bacteria and Tumor).

**Table 1:** Train/validation/test split for ImmunoDB.

	Train		Validation		Test	
	+	−	+	−	+	−
Virus	1464	1310	211	185	403	391
Bacteria	661	1071	78	169	174	322
Tumor	212	331	27	51	61	95

**Toxicity:** We utilized ToxDL 2.0 [26] dataset, a comprehensive toxicity database containing labeled toxic and non-toxic proteins. This dataset is split into two half according to the date of collection. The first part consists of proteins collected before January 1, 2022:

- **Training Set:** containing **4,879** toxic and **9,637** non-toxic proteins.
- **Validation Set:** containing **76** toxic and **837** non-toxic proteins.
- **Test Set:** containing **110** toxic and **1,696** non-toxic proteins.
  The second split of the data consists of proteins collected after the date of January 1, 2022:
- **Independent Set:** containing **152** toxic and **4,547** non-toxic proteins.

The original dataset has few more labeled very long protein sequences, which we excluded from our utilized dataset due to resource constraints while predicting protein structures using ESMFold.

### Experimental Settings

Each ensemble member $M_{k}$ was independently optimized. Separate ensembles were trained for each specific dataset:

- **Three** for the ImmunoDB dataset, one for each of the Immuno-Virus, Immuno-Bacteria, and Immuno-Tumor datasets.
- **One** for the toxicity dataset.

For MCD implementations, we used a dropout rate of 0.1, and only applied it before the final classification head linear layer. For DVBLL, LA, and DKL, we altered the original VenusVaccine architecture by inserting an additional linear layer into the final MLP segment. Only this new layer functions as the probabilistic segment, to achieve consistent training behavior with minimal computational burden, and its dimensions are 64, 64 and 16 for LA, DVBLL and DKL accordingly.

We obtain the probabilistic prediction $P_{M_{k}}$ through 64 MC sample predictions. For the deterministic baselines, we utilized deterministic VenusVaccine [11] model with protein sequence embeddings from 5 different PLMs.

### Results

All experiments conducted in this work can be summarized as follows:

- We compared our proposed **DUNE** approach with three deterministic ensemble baselines: *Majority Voting (MVote)*, *Soft Voting (SVote)* and *Performance Weighting (PWeight)*, one uncertainty based probabilistic ensemble baseline: *Uncertainty Voting (UVote)*, and also the deterministic single learner.
- We compared the three uncertainty weighting schemes: (i) *KL-Div (KLD)*, (ii) *Negative-softmax (NS)* and (iii) *Unbiased weighting (Unbiased)*, discussed in the **DUNE** subsection of the **Method** section.
- We compared several different BNNs in our proposed DUNE approach, evaluating both their predictive and UQ performances.

#### Experiment 1. Comparison with Baselines

Table 2 shows the comparative results on immunogenic virus, bacteria, tumor datasets, along with toxicity datasets. For DUNE method, we reported the combinations of weighting strategy and BNN that produced the highest accuracy and highest AUC-ROC. For UVote method, we reported the BNN that produced the highest accuracy and the highest AUC-ROC. For the single learner method, we reported those PLM embeddings for different datasets that achieved the highest accuracy among five different PLM embeddings. Following the approach mentioned in *PWeight* [40], we measured the member-specific weights by training a linear regression model on the validation dataset predictions.

**Table 2:** Results on protein property datasets.

Dataset	Method	BNN	WS	Accuracy(↑)	Precision(↑)	Recall(↑)	F1-Score(↑)	AUC-ROC(↑)
Virus	MVote	–	–	0.9232	0.9171	0.9330	0.9250	0.9805
	SVote	–	–	0.9345	0.9249	0.9479	0.9363	0.9809
	PWeight	–	–	0.9332	0.9268	0.9429	0.9348	0.9806
	SL(Ankh)	–	–	0.9131	**0.9380**	0.8957	0.9164	0.9582
	UVote	DKL	–	0.9320	0.9246	0.9429	0.9337	0.9650
	UVote	SWAG	–	0.9282	0.9159	0.9454	0.9304	0.9713
	DUNE^*^	MCD	NS(c = 5)	**0.9395**	0.9298	**0.9529**	**0.9412**	0.9810
	DUNE^*^	MCD	NS(c = 25)	0.9383	0.9296	0.9504	0.9399	**0.9812**
Bacteria	MVote	–	–	0.8286	0.7987	0.6839	0.7368	0.8794
	SVote	–	–	0.8327	0.8054	0.6897	0.7430	0.8883
	PWeight	–	–	**0.8488**	0.8153	0.7356	**0.7734**	0.8851
	SL(Ankh)	–	–	0.8306	0.6954	**0.7961**	0.7423	0.8616
	UVote	MCD	–	0.8387	0.8052	0.7126	0.7561	0.8883
	DUNE^*^	MCD	Unbiased	**0.8448**	**0.8170**	0.7184	0.7645	0.8892
	DUNE^*^	DVBLL	NS(c = 25)	0.8347	0.7771	0.7414	0.7588	**0.8914**
Tumor	MVote	–	–	0.7436	0.6329	**0.8197**	0.7143	0.8336
	SVote	–	–	0.7500	0.6486	0.7869	0.7111	0.8483
	PWeight	–	–	0.7628	0.6765	0.7541	0.7132	0.8383
	SL(Ankh)	–	–	0.7692	**0.8852**	0.6506	**0.7500**	0.8507
	UVote	DVBLL	–	0.7756	0.6806	0.8033	0.7368	0.8214
	UVote	SWAG	–	0.7628	0.7069	0.6721	0.6891	0.8330
	DUNE^*^	DVBLL	NS(c = 25)	**0.7885**	0.7000	0.8033	0.7481	0.8373
	DUNE^*^	SWAG	NS(c = 5)	0.7628	0.6765	0.7541	0.7132	**0.8602**
Toxicity${}^{T}$	MVote	–	–	0.9845	0.8534	**0.9000**	0.8761	0.9896
	SVote	–	–	0.9845	0.8596	0.8909	0.8750	0.9901
	PWeight	–	–	**0.9856**	**0.8684**	**0.9000**	**0.8839**	0.9896
	SL(Ankh)	–	–	0.9801	0.8273	0.8426	0.8349	0.9858
	UVote	MCD	–	0.9845	0.8534	**0.9000**	0.8761	**0.9933**
	DUNE^*^	MCD	KLD	0.9850	0.8673	0.8909	0.8789	0.9904
	DUNE^*^	SWAG	KLD	0.9817	0.8235	0.8909	0.8559	**0.9933**
Toxicity${}^{I}$	MVote	–	–	0.9611	0.4378	0.7171	0.5436	0.9619
	SVote	–	–	0.9636	0.4603	**0.7237**	0.5627	0.9637
	PWeight	–	–	0.9664	0.4865	0.7105	0.5775	0.9630
	SL(ESM2)	–	–	0.9600	**0.7105**	0.4286	0.5347	0.9601
	UVote	DVBLL	–	0.9674	0.4974	0.6184	0.5513	0.9619
	UVote	MCD	–	0.9617	0.4426	0.7105	0.5455	**0.9688**
	DUNE^*^	DVBLL	KLD	**0.9698**	0.5269	0.6447	**0.5799**	0.9619
	DUNE^*^	MCD	Unbiased	0.9625	0.4496	0.7039	0.5487	**0.9688**

*MVote*, *SVote*, *PWeight* and *SL* refers to majority voting, soft voting, performance weighting and single learner methods accordingly. KLD denotes KL-Divergence weighting and NS denotes Negative-Softmax. Toxicity${}^{T}$ and Toxicity${}^{I}$ denote the test and independent toxicity datasets accordingly. WS denotes the weighting strategy used for DUNE method. For each dataset and for each metric, the optimal value is **boldfaced** and the second optimal value is underlined.

Even though the same combination of BNN and weighting strategy did not obtain the optimal accuracy and AUC-ROC for all datasets, our proposed DUNE model outperformed the baselines at majority of the metrics across all datasets, achieving either the best or the second best performance.

DUNE obtained the best performance across all metrics for immunogenic virus dataset, except precision where it shows the second best performance. However, the baseline method achieving best precision lagged behind DUNE in terms of all the other metrics.

DUNE obtained higher accuracy, precision and AUC-ROC for immunogenic bacteria dataset while delivering the second best performance on other metrics. Notably, it offers the best and the second best AUC-ROC. The baseline PWeight method obtained similar accuracy, higher F1-score, but lower AUC-ROC than both DUNE combinations reported in Table 2.

DUNE obtained high accuracy and AUC-ROC for immunogenic tumor dataset, while offering the second best performance on nearly all other metrics but precision where it just slightly lagged behind UVote, by less than 0.007, that offers the second best performance. Unlike the observation on all other datasets, the single learner baseline obtained consistent results for immunogenic tumor dataset.

Compared to all the rest of the datasets, DUNE primarily lagged behind the other baselines across all metrics, offering the second best performances only by very small margins, with less than 0.001 worse performance in Accuracy, Precision, and less than 0.01 in Recall and F1 score for test toxicity dataset.

For independent toxicity dataset, DUNE outperformed baselines in terms of accuracy, F1-score and AUC-ROC. With respect to Precision, single learner provided the best performance while DUNE offered the second best performance. Only with respect to Recall, DUNE marginally underperformed the baselines (less than 0.02−0.01 with respect to best and second best performances).

In summary, DUNE outperformed all deterministic and probabilistic baselines in terms of most metrics and for all datasets, however not with one single combination of BNN and weighting strategy.

#### Experiment 2. Comparative Evaluation of Uncertainty Weighting Approaches

Table 3 shows the comparative results on different weighting approaches. We used MCD based BNN to convert the ensemble members into probabilistic models and reported the corresponding results. Results for other BNNs have been reported in supplementary material.

**Table 3:** Comparative results on different weighting approaches.

Dataset	WS	Accuracy(↑)	Precision(↑)	Recall(↑)	F1 Score(↑)	AUC ROC(↑)	NLL(↓)
Virus	KLD	0.9358	0.9231	**0.9529**	0.9377	0.9804	**0.1788**
	NS(c = 5)	**0.9395**	**0.9298**	**0.9529**	**0.9412**	0.9810	0.1836
	NS(c = 25)	0.9383	0.9296	0.9504	0.9399	**0.9812**	0.1794
	NS(c = 100)	0.9370	0.9253	**0.9529**	0.9389	0.9807	0.1803
	Unbiased	0.9282	0.9159	0.9454	0.9304	0.9711	0.4163
Bacteria	KLD	0.8367	0.8039	0.7069	0.7523	**0.8896**	0.5159
	NS(c = 5)	0.8327	0.8054	0.6897	0.7430	0.8883	**0.4800**
	NS(c = 25)	0.8327	0.8054	0.6897	0.7430	0.8881	0.4880
	NS(c = 100)	0.8407	0.8105	0.7126	0.7584	0.8881	0.5245
	Unbiased	**0.8448**	**0.8170**	**0.7184**	**0.7645**	0.8892	0.8412
Tumor	KLD	**0.7564**	**0.6716**	0.7377	0.7031	0.8378	0.5916
	NS(c = 5)	0.7436	0.6400	**0.7869**	**0.7059**	**0.8475**	**0.4718**
	NS(c = 25)	0.7308	0.6338	0.7377	0.6818	**0.8475**	0.4822
	NS(c = 100)	0.7436	0.6522	0.7377	0.6923	0.8385	0.5734
	Unbiased	0.7500	0.6618	0.7377	0.6977	0.8264	1.7150
Toxicity${}^{T}$	KLD	**0.9850**	**0.8673**	0.8909	**0.8789**	0.9904	**0.0515**
	NS(c = 5)	0.9845	0.8596	0.8909	0.8750	0.9902	0.0534
	NS(c = 25)	0.9845	0.8596	0.8909	0.8750	0.9903	0.0518
	NS(c = 100)	0.9839	0.8462	**0.9000**	0.8722	0.9908	0.0520
	Unbiased	0.9839	0.8462	**0.9000**	0.8722	**0.9931**	0.1631
Toxicity${}^{I}$	KLD	**0.9645**	**0.4681**	**0.7237**	**0.5685**	0.9648	0.1003
	NS(c = 5)	0.9634	0.4580	0.7171	0.5590	0.9634	0.0979
	NS(c = 25)	0.9638	0.4619	0.7171	0.5619	0.9631	**0.0969**
	NS(c = 100)	0.9636	0.4599	0.7171	0.5604	0.9646	0.1041
	Unbiased	0.9625	0.4496	0.7039	0.5487	**0.9683**	0.2620

KLD denotes KL-Divergence weighting and NS denotes Negative-Softmax weighting. All reported results for DUNE methods with each member as a MCD based probabilistic model. KLD denotes KL-Divergence weighting and NS denotes Negative-Softmax. Toxicity${}^{T}$ and Toxicity${}^{I}$ denote the test and independent toxicity datasets accordingly. WS denotes the weighting strategy used for DUNE method. For each dataset and for each metric, the optimal value is **boldfaced** and the second optimal value is underlined.

KLD weighting performed better than other weighting approaches for immunogenic tumor dataset and also for both test and independent toxicity datasets. Also, in terms of those metrics where KLD lagged behind other weighting schemes, the difference was minimal. Specifically, KLD performed in most consistent manner for both of the toxicity datasets.

NS weighting with c=5 performed better than other approaches for immunogenic virus dataset across most of the metrics. For immunogenic tumor dataset, unbiased weighting approach outperformed other approaches, specially in terms of the predictive metrics. Unbiased weighting obtained high NLL compared to other approaches for all the datasets, indicating low confidence in its predictions (providing predicted probabilities at the correct side of the the decision boundary, 0.5, but distant from the true labels, either 0 or 1).

For the NS-based weighting, smaller values of the control parameter yielded better performance than larger values in majority cases. This aligns with expectations, as higher control parameter values bias the model towards UVote approach.

In summary, KLD obtained a more consistent performance for most datasets and across all predictive and UQ metrics. NS weighting approach with smaller *c* values obtained better performance than with higher *c* values, indicating the superiority of uncertainty based weighting ensemble over UVote based ensemble method.

#### Experiment 3. Comparative Probabilistic Member Architectures

Apart from MCD, we also tested other probabilistic deep model architectures in the proposed DUNE method. Table 4 shows the results for different probabilistic models with KLD based weighting approach for DUNE method. Results for other weighting strategies have been reported in supplementary material.

**Table 4:** Comparative results for different probabilistic ensemble member model.

Prediction Metric	Model	Virus	Bacteria	Tumor	Toxicity${}^{T}$	Toxicity${}^{I}$
Accuracy(↑)	MCD	**0.9358**	0.8367	0.7564	**0.9850**	0.9645
	DKL	0.9332	**0.8387**	0.6795	0.9845	0.9640
	DVBLL	0.9332	0.8327	**0.7756**	0.9845	**0.9698**
	LA	0.9232	0.8286	0.7500	0.9845	0.9668
	SWAG	0.9345	0.8347	0.7500	0.9817	0.9608
Precision(↑)	MCD	0.9231	0.8039	0.6716	**0.8673**	0.4681
	DKL	0.9227	**0.8092**	0.5965	0.8661	0.4612
	DVBLL	**0.9375**	0.7725	**0.6857**	0.8596	**0.5269**
	LA	0.9233	0.7572	0.6447	0.8661	0.4896
	SWAG	0.9249	0.7738	0.6667	0.8235	0.4380
Recall(↑)	MCD	**0.9529**	0.7069	0.7377	**0.8909**	0.7237
	DKL	0.9479	0.7069	0.5574	0.8818	0.6645
	DVBLL	0.9305	0.7414	0.7869	**0.8909**	0.6447
	LA	0.9256	**0.7529**	**0.8033**	0.8818	0.6184
	SWAG	0.9479	0.7471	0.7213	**0.8909**	**0.7434**
F1 Score(↑)	MCD	**0.9377**	0.7523	0.7031	**0.8789**	0.5685
	DKL	0.9351	0.7546	0.5763	0.8739	0.5445
	DVBLL	0.9340	0.7566	**0.7328**	0.8750	**0.5799**
	LA	0.9244	0.7550	0.7153	0.8739	0.5465
	SWAG	0.9363	**0.7602**	0.6929	0.8559	0.5512
AUC-ROC(↑)	MCD	**0.9804**	0.8896	0.8378	0.9904	**0.9648**
	DKL	0.9771	0.8750	0.7220	0.9899	0.9506
	DVBLL	0.9762	**0.8916**	**0.8502**	0.9907	0.9619
	LA	0.9749	0.8858	0.8380	0.9904	0.9591
	SWAG	0.9792	0.8796	0.8373	**0.9932**	0.9633

Toxicity${}^{T}$ and Toxicity${}^{I}$ denote the test and independent toxicity datasets accordingly. All reported results are for DUNE method with KLD based weighting strategy. For each dataset and for each metric, the optimal value is **boldfaced** and the second optimal value is underlined.

In terms of predictive UQ performance, different BNNs exhibited varying strengths across different datasets. Overall, MCD and DVBLL performed better than their counterparts at majority of metrics across all datasets. MCD performed in a better manner across Virus and test toxicity datasets, and DVBLL performed in a better manner in Tumor and independent toxicity datasets. For Bacteria dataset, different BNNs showed superior performance across different metrics, with SWAG being the most consistent candidate despite failing to achieve the best value across all metrics.

In summary, even though no single BNN obtained optimal performance across all datasets, one single BNN obtained optimal performance for a specific dataset across most of the metrics.

#### Experiment 4. Uncertainty Quantification Evaluation

We evaluated different probabilistic deep model architectures for uncertainty quantification. Table 5 shows the results for different probabilistic models on several uncertainty quantification metrics, along with our proposed Cross-Divergence metric with KLD based weighting approach. Results for other weighting strategies have been reported in supplementary material.

**Table 5:** Uncertainty quantification evaluation.

UQ Metric	Model	Virus	Bacteria	Tumor	Toxicity${}^{T}$	Toxicity${}^{I}$
CDiv(↑)	MCD	**6.6719**	0.5928	−3.6989	12.4150	9.8370
	DKL	3.5264	0.2320	0.0006	6.6525	6.1032
	DVBLL	5.6940	−5.5328	−0.6190	11.5257	10.3730
	LA	6.1067	0.4271	−1.8109	**13.4206**	**10.8600**
	SWAG	4.7570	**2.2087**	**1.6627**	8.5277	7.4408
ECE(↓)	MCD	**0.0060**	0.0964	0.1242	0.0057	0.0285
	DKL	0.0470	0.1716	0.1782	0.0362	0.0540
	DVBLL	0.0153	0.1118	0.1060	**0.0030**	**0.0185**
	LA	0.0154	0.0940	0.1270	0.0031	0.0187
	SWAG	0.0370	**0.0476**	**0.0742**	0.0182	0.0508
NLL(↓)	MCD	**0.1788**	0.5159	0.5916	**0.0515**	0.1003
	DKL	0.2140	0.5049	0.6917	0.0784	0.1160
	DVBLL	0.2012	0.5624	0.5393	0.0534	**0.0860**
	LA	0.2014	0.4784	0.5708	0.0551	0.0961
	SWAG	0.1958	**0.4200**	**0.4960**	0.0600	0.1099
Brier Score(↓)	MCD	**0.0496**	0.1345	0.1905	**0.0130**	0.0284
	DKL	0.0544	0.1597	0.2493	0.0141	0.0270
	DVBLL	0.0541	0.1359	0.1747	0.0141	**0.0236**
	LA	0.0576	0.1353	0.1849	0.0142	0.0258
	SWAG	0.0531	**0.1294**	**0.1655**	0.0147	0.0305

Toxicity${}^{T}$ and Toxicity${}^{I}$ denote the test and independent toxicity datasets accordingly. All reported results are for DUNE method with KLD based weighting strategy. For each dataset and for each metric, the optimal value is **boldfaced** and the second optimal value is underlined.

These results demonstrate that our proposed Cross-Divergence (CDiv) uncertainty quantification (UQ) metric generally aligns with other established UQ metrics, specially in Virus, Bacteria and Tumor datasets, where MCD performed best in Virus datasets at all metrics and SWAG performed best in Bacteria and Tumor datasets.

In test toxicity dataset, no one model provided optimum values across all four metrics. But in general, MCD, DVBLL and LA achieved low ECE, NLL, Brier Score and high CDiv. In independent toxicity dataset, DVBLL yielded optimal results for Expected Calibration Error (ECE), Negative Log-Likelihood (NLL), and Brier Score, while LA performed best according to CDiv. However, in general all four metrics behaved quite coherently for both DVBLL and LA.

In summary, our proposed CDiv metric’s behavior mostly aligned with other UQ metrics across all datasets. Similar to the scenario in terms of predictive metrics, no single BNN achieved optimal performance across all datasets in terms of UQ metrics. In general, MCD and SWAG obtained better performances in all immunogenicity datasets, and MCD and DVBLL obtained better performances in both toxicity datasets. Further investigation into the characteristic of CDiv is discussed in Section **Investigative Study on Utility of Cross-Divergence**.

### Investigative Study on Utility of Uncertainty Estimates into Ensemble

We further investigate the utility of uncertainty estimates into deep ensemble methods. We report the uncertainty estimates (σ’s from probabilistic model prediction $N(\mu ,\sigma^{2})$) of different probabilistic models. We also categorize those uncertainty estimates according to four classification categories: TP(True Positives), TN(True Negatives), FP(False Positives) and FN(False Negatives). Table 6 shows the uncertainty estimates of those probabilistic models that utilize the ESM-Cambrian [48] protein language model embeddings for sequence level protein representation.

**Table 6:** Uncertainty estimates, σ’s, of different probabilistic models.

Dataset	Model	TP	TN	FP	FN
Virus	MCD	$0.0055_{\pm 0.0092}$	$0.0043_{\pm 0.0104}$	$0.0237_{\pm 0.0179}$	$0.0216_{\pm 0.0170}$
	DKL	$0.1001_{\pm 0.0217}$	$0.1150_{\pm 0.0239}$	$0.1287_{\pm 0.0323}$	$0.1342_{\pm 0.0308}$
	DVBLL	$0.0249_{\pm 0.0363}$	$0.0116_{\pm 0.0282}$	$0.0805_{\pm 0.0491}$	$0.0641_{\pm 0.0464}$
	LA	$0.0074_{\pm 0.0072}$	$0.0053_{\pm 0.0073}$	$0.0184_{\pm 0.0071}$	$0.0164_{\pm 0.0074}$
	SWAG	$0.1108_{\pm 0.0527}$	$0.1154_{\pm 0.0494}$	$0.1721_{\pm 0.0440}$	$0.1631_{\pm 0.0501}$
Bacteria	MCD	$0.0043_{\pm 0.0077}$	$0.0035_{\pm 0.0080}$	$0.0139_{\pm 0.0139}$	$0.0090_{\pm 0.0091}$
	SVDKL	$0.1447_{\pm 0.0099}$	$0.1213_{\pm 0.0115}$	$0.1385_{\pm 0.0101}$	$0.1287_{\pm 0.0151}$
	DVBLL	$0.0581_{\pm 0.0509}$	$0.0322_{\pm 0.0452}$	$0.1049_{\pm 0.0482}$	$0.0754_{\pm 0.0567}$
	LA	$0.0147_{\pm 0.0155}$	$0.0093_{\pm 0.0146}$	$0.0278_{\pm 0.0207}$	$0.0186_{\pm 0.0178}$
	SWAG	$0.1446_{\pm 0.0580}$	$0.1310_{\pm 0.0525}$	$0.1867_{\pm 0.0537}$	$0.1644_{\pm 0.0516}$
Tumor	MCD	$0.0149_{\pm 0.0027}$	$0.0090_{\pm 0.0054}$	$0.0150_{\pm 0.0020}$	$0.0145_{\pm 0.0025}$
	DKL	$0.0267_{\pm 0.0008}$	$0.0269_{\pm 0.0013}$	$0.0268_{\pm 0.0011}$	$0.0263_{\pm 0.0012}$
	DVBLL	$0.1349_{\pm 0.0282}$	$0.0754_{\pm 0.0490}$	$0.1382_{\pm 0.0313}$	$0.1320_{\pm 0.0373}$
	LA	$0.0543_{\pm 0.0221}$	$0.0264_{\pm 0.0248}$	$0.0460_{\pm 0.0239}$	$0.0558_{\pm 0.0244}$
	SWAG	$0.1151_{\pm 0.0143}$	$0.0581_{\pm 0.0422}$	$0.1198_{\pm 0.0113}$	$0.0970_{\pm 0.0243}$
Toxicity${}^{T}$	MCD	$0.0084_{\pm 0.0225}$	$0.0009_{\pm 0.0071}$	$0.0314_{\pm 0.0345}$	$0.0303_{\pm 0.0245}$
	DKL	$0.0812_{\pm 0.0405}$	$0.0346_{\pm 0.0267}$	$0.1246_{\pm 0.0507}$	$0.1350_{\pm 0.0546}$
	DVBLL	$0.0153_{\pm 0.0210}$	$0.0009_{\pm 0.0062}$	$0.0340_{\pm 0.0238}$	$0.0171_{\pm 0.0219}$
	LA	$0.0022_{\pm 0.0017}$	$0.0001_{\pm 0.0006}$	$0.0033_{\pm 0.0012}$	$0.0023_{\pm 0.0020}$
	SWAG	$0.1792_{\pm 0.0619}$	$0.0388_{\pm 0.0449}$	$0.1919_{\pm 0.0583}$	$0.1740_{\pm 0.0810}$
Toxicity${}^{I}$	MCD	$0.0181_{\pm 0.0364}$	$0.0022_{\pm 0.0111}$	$0.0354_{\pm 0.0387}$	$0.0360_{\pm 0.0431}$
	DKL	$0.0933_{\pm 0.0465}$	$0.0402_{\pm 0.0347}$	$0.1223_{\pm 0.0504}$	$0.1059_{\pm 0.0585}$
	DVBLL	$0.0175_{\pm 0.0230}$	$0.0017_{\pm 0.0086}$	$0.0332_{\pm 0.0227}$	$0.0165_{\pm 0.0241}$
	LA	$0.0020_{\pm 0.0015}$	$0.0002_{\pm 0.0008}$	$0.0034_{\pm 0.0014}$	$0.0022_{\pm 0.0019}$
	SWAG	$0.1753_{\pm 0.0646}$	$0.0505_{\pm 0.0593}$	$0.2179_{\pm 0.0687}$	$0.1725_{\pm 0.0710}$

TP, TN, FP, FN denote true positive, true negative, false positive, and false negative accordingly. Each element represents uncertainty estimates over the entire test set in a mean ± standard deviation format.

For most probabilistic models, and also for most of the datasets, we see that the uncertainty estimates, σ’s, of true positive and true negative predictions are usually lower than false positive and false negative predictions. This observation indicates that for a single data point prediction, if we set higher weights for members with low uncertainty estimates and lower weights for members with higher uncertainty estimates, we will effectively reduce the effect of a wrong prediction from a member in that ensemble.

For immunogenic tumor and independent toxicity dataset, the average uncertainty estimates, σ’s, are higher for correct predictions than incorrect predictions for some of the probabilistic models. This phenomenon explains the overall suboptimal performances of our proposed models for these two specific datasets, compared to the rest of the datasets.

In summary, we observe that the predicted uncertainties for correct predictions (TP, TN) are primarily lower than incorrect predictions (FP, FN) for most of the different probabilistic models utilized in this work and also across most of the datasets. This observation validates the empirical results for the superior performance of uncertainty weighted ensemble methods over the deterministic ensemble baselines.

Even though most of the probabilistic models show low uncertainty estimates for correct predictions and vice versa, some scenarios show opposite outcome. Also, most of the existing UQ metrics, specifically the three deployed in this work (ECE, NLL and Brier Score) are not sensitive to prediction variabilities or uncertainty estimates, σ’s. We investigate our proposed prediction variability sensitive novel metric, Cross-Divergence’s utility to quantify such uncertainties in the next Section.

### Investigative Study on Utility of Cross-Divergence

We further investigate the utility of our proposed UQ metric: Cross-Divergence and its sensitive nature to predicted uncertainty estimates of probabilistic models, σ’s. Table 7 reports the uncertainty estimate of each ensemble member of different DUNE models with different BNN. The reported results are for Virus dataset, and the DUNE models employ KL-Divergence based weighting strategy.

**Table 7:** Uncertainty estimates, σ’s, of different ensemble members, and evaluated UQ metrics for different BNNs in DUNE setting.

Model	PLM	TP	TN	FP	FN	CDiv(↑)	ECE(↓)	NLL(↓)	Brier Score(↓)
MCD	ESMC	$0.0055_{\pm 0.0092}$	$0.0043_{\pm 0.0104}$	$0.0237_{\pm 0.0179}$	$0.0216_{\pm 0.0170}$	**6.6719**	**0.0060**	**0.1788**	**0.0496**
	ProstT5	$0.0017_{\pm 0.0047}$	$0.0019_{\pm 0.0055}$	$0.0089_{\pm 0.0095}$	$0.0122_{\pm 0.0123}$				
	Ankh	$0.0016_{\pm 0.0058}$	$0.0021_{\pm 0.0064}$	$0.0135_{\pm 0.0147}$	$0.0111_{\pm 0.0149}$				
	ESM2	$0.0078_{\pm 0.0066}$	$0.0060_{\pm 0.0083}$	$0.0199_{\pm 0.0058}$	$0.0159_{\pm 0.0061}$				
	Prot Bert	$0.0037_{\pm 0.0075}$	$0.0029_{\pm 0.0055}$	$0.0131_{\pm 0.0093}$	$0.0122_{\pm 0.0094}$				
DKL	ESMC	$0.1001_{\pm 0.0217}$	$0.1150_{\pm 0.0239}$	$0.1287_{\pm 0.0323}$	$0.1342_{\pm 0.0308}$	3.5264	0.0470	0.2140	0.0544
	ProstT5	$0.0411_{\pm 0.0321}$	$0.0447_{\pm 0.0440}$	$0.1049_{\pm 0.0769}$	$0.0967_{\pm 0.0712}$				
	Ankh	$0.1205_{\pm 0.0272}$	$0.1226_{\pm 0.0191}$	$0.1513_{\pm 0.0307}$	$0.1433_{\pm 0.0237}$				
	ESM2	$0.0327_{\pm 0.0351}$	$0.0312_{\pm 0.0340}$	$0.0801_{\pm 0.0655}$	$0.0744_{\pm 0.0710}$				
	Prot Bert	$0.0765_{\pm 0.0321}$	$0.0932_{\pm 0.0330}$	$0.1185_{\pm 0.0587}$	$0.1239_{\pm 0.0495}$				
DVBLL	ESMC	$0.0249_{\pm 0.0363}$	$0.0116_{\pm 0.0282}$	$0.0805_{\pm 0.0491}$	$0.0641_{\pm 0.0464}$	5.6940	0.0153	0.2012	0.0541
	ProstT5	$0.0126_{\pm 0.0278}$	$0.0091_{\pm 0.0260}$	$0.0535_{\pm 0.0464}$	$0.0596_{\pm 0.0521}$				
	Ankh	$0.0107_{\pm 0.0297}$	$0.0085_{\pm 0.0296}$	$0.0701_{\pm 0.0639}$	$0.0402_{\pm 0.0608}$				
	ESM2	$0.0095_{\pm 0.0335}$	$0.0077_{\pm 0.0350}$	$0.0517_{\pm 0.0636}$	$0.0780_{\pm 0.0839}$				
	Prot Bert	$0.0258_{\pm 0.0262}$	$0.0271_{\pm 0.0305}$	$0.0599_{\pm 0.0289}$	$0.0752_{\pm 0.0209}$				
LA	ESMC	$0.0074_{\pm 0.0072}$	$0.0053_{\pm 0.0073}$	$0.0184_{\pm 0.0071}$	$0.0164_{\pm 0.0074}$	6.1067	0.0154	0.2014	0.0576
	ProstT5	$0.0019_{\pm 0.0047}$	$0.0020_{\pm 0.0044}$	$0.0099_{\pm 0.0071}$	$0.0090_{\pm 0.0066}$				
	Ankh	$0.0006_{\pm 0.0018}$	$0.0003_{\pm 0.0016}$	$0.0033_{\pm 0.0046}$	$0.0027_{\pm 0.0036}$				
	ESM2	$0.0007_{\pm 0.0023}$	$0.0008_{\pm 0.0026}$	$0.0036_{\pm 0.0044}$	$0.0045_{\pm 0.0049}$				
	Prot Bert	$0.0084_{\pm 0.0057}$	$0.0074_{\pm 0.0071}$	$0.0143_{\pm 0.0063}$	$0.0130_{\pm 0.0043}$				
SWAG	ESMC	$0.1108_{\pm 0.0527}$	$0.1154_{\pm 0.0494}$	$0.1721_{\pm 0.0440}$	$0.1631_{\pm 0.0501}$	4.7570	0.0370	0.1958	0.0531
	ProstT5	$0.1162_{\pm 0.0702}$	$0.1072_{\pm 0.0892}$	$0.2019_{\pm 0.0779}$	$0.2006_{\pm 0.0933}$				
	Ankh	$0.0897_{\pm 0.0777}$	$0.0647_{\pm 0.0675}$	$0.2220_{\pm 0.0754}$	$0.1427_{\pm 0.0928}$				
	ESM2	$0.1020_{\pm 0.0666}$	$0.1084_{\pm 0.0727}$	$0.1848_{\pm 0.0523}$	$0.1953_{\pm 0.0673}$				
	Prot Bert	$0.1186_{\pm 0.0674}$	$0.1031_{\pm 0.0796}$	$0.2136_{\pm 0.0630}$	$0.1797_{\pm 0.0699}$				

For all models, KLD based weighting strategy is deployed.

We observe that each ensemble member of DUNE method for MCD and LA show low uncertainty estimates, σ’s, in general, and obtained high CDiv as a result. Also the uncertainty estimates, σ’s, for correct predictions (TP, TN) on average are multiple times lower in magnitude than for incorrect predictions (FP, FN).

Even though MCD obtained the optimal performance according to all the UQ metrics, the second most optimal performer varied across different metrics. LA models obtained low uncertainty estimates overall, and also obtained even lower uncertainty estimates for correct predictions than incorrect predictions compared to other BNNs (except MCD). As a result, LA obtained second to best value for Cross-Divergence metric. Also, DKL obtained the lowest value for Cross-Divergence even though it obtained better accuracy than LA (Table 4). This observation can be attributed to the fact that the difference between uncertainty estimates for correct and incorrect predictions in DKL based DUNE model is generally lower than for LA based DUNE model.

In summary, we can deduce that probabilistic models that typically show low uncertainty estimates for correct predictions and high uncertainty estimates for incorrect predictions, and a visibly high difference between uncertainty estimates for correct and incorrect predictions, tend to obtain high Cross-Divergence.

### Case Study

As a case study, we evaluate the effectiveness of the DUNE method in identifying clinically validated vaccine targets within the SARS-CoV-2 proteome. We perform a re-discovery experiment with the SARS-CoV-2 case study dataset used in [11], which contains 26 sequences from the SARS-CoV-2 Data Hub [55], along with the most important protein: surface glycoprotein (NCBI ID: YP 009724390.1), considering the fact that surface glycoprotein is the primary antigen recognized by the immune system [56].

We rank all these 26 proteins according to the predicted probabilities for the positive class for both deterministic and probabilistic models employed in this work. Table 8 shows the rank of surface glycoprotein for different probabilistic and deterministic models. Each column shows ranks for different models with protein representations from different protein language models.

**Table 8:** Rank of surface glycoprotein (NCBI ID: YP 009724390.1).

Model	ESMC	ProstT5	Ankh	ESM2	Prot Bert
Deterministic	16	**1**	**1**	4	**1**
MCD	16	**1**	**1**	4	**1**
DKL	**1**	5	**1**	5	3
DVBLL	4	**1**	**1**	4	**1**
LA	3	2	2	2	**1**
SWAG	3	3	**1**	**1**	**1**

We observe that, even though deterministic models with protein representations from ProstT5, Ankh and Prot Bert model rank the surface glycoprotein at top, rest of the two models do not rank them at top. In fact, the deterministic model based on the protein representation from ESMC ranks the surface glycoprotein at $16^{th}$ position. All probabilistic models, in general, rank the surface glycoprotein high. Most notably, probabilistic models (except Monte-Carlo Dropout) with the protein representation from ESMC ranks the surface glycoprotein much higher than what their deterministic counterpart does.

We further evaluate DUNE for different BNNs and for different weighting strategies over this 26 proteins and report the rank of surface glycoprotein and the predicted positive class probability in Table 9.

**Table 9:** Rank and predicted positive class probability of surface glycoprotein (NCBI ID: YP 009724390.1) for DUNE models with different weighting strategies and for different BNNs.

Model	DUNE									
	KLD		NS(c = 5)		NS(c = 25)		NS(c = 100)		Unbiased	
	$\hat{r}$	$\hat{p}$	$\hat{r}$	$\hat{p}$	$\hat{r}$	$\hat{p}$	$\hat{r}$	$\hat{p}$	$\hat{r}$	$\hat{p}$
MCD	1	0.981541	3	0.891495	1	0.941645	1	0.985176	2	0.999979
DKL	2	0.912184	1	0.871254	4	0.950095	5	0.970417	4	0.955200
DVBLL	2	0.978312	1	0.967587	1	0.983123	3	0.997355	4	0.999977
LA	2	0.967910	1	0.940030	1	0.947437	1	0.970159	2	0.999995
SWAG	2	0.939219	1	0.937265	3	0.940825	3	0.946340	3	0.940419

$\hat{r}$ and $\hat{p}$ indicates the rank and predicted probability accordingly.

Table 9 shows that DUNE in general ranks the surface glycoprotein at a high position for different BNNs and also with different weighting strategies. Among different BNNs, MCD and LA rank the surface glycoprotein at the top position at majority scenarios with different weighting strategies. Among different weighting strategies, KLD and NS with c=5 rank the surface glycoprotein at the top or second to top position in a consistent manner for different BNNs. Even though the Unbiased weighting strategy does not rank the surface glycoprotein at the top position with any BNN, the predicted positive class probability is in general much higher for Unbiased weighting strategy than other weighting strategies.

## Conclusion

Advancing data-driven and computationally intensive methods for evaluating protein properties is crucial for developing immunogenic therapeutics such as vaccines, where safety and efficacy are paramount. The rise of machine learning (ML) has opened new avenues in this field. When public health is at stake, uncertainty quantification (UQ) integrated in these AI/ML methods becomes vital, not just to gauge prediction reliability, but also to increase predictive performance. Our research introduces a novel methodology that enhances protein property prediction by integrating uncertainty quantification into deep ensemble models. We also propose a novel UQ metric specifically designed for evaluating probabilistic deep learning classifiers. Experimental evaluations indicate that integration of uncertainty estimates of probabilistic models into deep ensemble methods achieve superior performance than single learners and other deterministic and probabilistic deep ensemble approaches. Apart from straightforward evaluation, we also investigate the utility of uncertainty estimates into deep ensemble approaches and capability of our proposed DUNE method to find clinically validated vaccine targets. Our approach focuses on epistemic uncertainty to achieve enhanced predictive performance. We recognize that addressing uncertainty in protein representation and its impact on prediction remains an important area for future research.

## Uncertainty Evaluation Metrics

The three established UQ metrics we utilized in this work are Expected Calibration Error (ECE), Negative Log-Likelihood (NLL), and the Brier Score, which have been commonly employed in the literature. ECE and Brier scores are considered to assess a model’s calibration, indicating how well its predicted probabilities align with the true likelihood of events, while NLL is mostly regarded as an indicator of overconfidence, revealing when a model is overly certain about its predictions, even if they are incorrect.

### Expected Calibration Error (ECE)

ECE partitions predictions into *M* equally-spaced bins based on their prediction confidence, ECE can be calculated as,


(17) $$\mathrm{ECE}=\sum_{m=1}^{M}\frac{B_{m}}{N}|\text{acc}(B_{m})-\text{conf}(B_{m})|$$

with *N* indicating the size of the dataset, and $\text{acc}(B_{m})=1/|B_{m}|$  $\sum_{i\in B_{m}}I({\tilde{y}}_{i}=y_{i})$ and $\text{conf}(B_{m})=1/|B_{m}|\sum_{i\in B_{m}}{\hat{y}}_{i}$ the average accuracy and confidence in bin $B_{m}$ with size $\left|B_{m}\right|$ accordingly. Here, $\tilde{y}$ is the predicted label and $\hat{y}$ is the predicted class probability. For, binary classification problem, $\tilde{y}=I(\hat{y}\geq \theta_{d})$, with $\theta_{d}$ as the decision threshold, usually 0.5 for binary classification problem.

### Brier Score

For a binary classification, this metric is evaluated as:


(18) $$\mathrm{Brier}=\frac{1}{N}\sum_{i=1}^{N}{\left(y_{i}-{\hat{y}}_{i}\right)}^{2}$$

Here *y* denotes the true label and $\hat{y}$ denotes the predicted positive class probability.

### Negative Log-Likelihood

Negative log-likelihood is computed as the negative log-probability assigned to the true label,


(19) $$\mathrm{NLL}(x)=-y(x)\;\text{log}(\hat{y}(x))-(1-y(x))\;\text{log}(1-\hat{y}(x))$$

When a model is overconfident in an incorrect prediction, it assigns a high probability to the wrong class. Therefore, the log loss becomes very large, that results in a high NLL.

## Protein Language Models

We provide brief description for each of the protein language models utilized for embedding extraction in this work:

**ProstT5:** This is a bilingual PLM, based on encoder-decoder T5 [57] model, that is fine-tuned with the goal to translate between amino acids and structural 3Di tokens—introduced by Foldseek [52]. Particularly, it is trained on a non-redundant subset of high confidence protein structures from AlphaFold structure Database (AFDB) [58]. The extracted sequence and structure embeddings from the encoder then can be used in downstream tasks.

**Ankh:** A family of encoder-only Protein Language Models (PLMs) designed for compute efficiency and strong generalization. It follows an ESM-2-like architecture, is pretrained on UniRef50 [59] using masked language modeling (MLM), and scales from 5M to 650M parameters. Despite its smaller size, Ankh matches or outperforms larger models like ESM-2 and xTrimoPGLM [60] by adhering to compute-optimal scaling laws. The authors show that performance does not scale linearly with size—well-optimized small models can be more effective than their larger counterparts.

**ESM-2:** A large-scale transformer-based Protein Language Model (PLM) trained with a masked language modeling (MLM) objective on evolutionary-scale sequence data, scaling up to 15 billion parameters. It directly predicts atomic-level 3D structures from primary sequences, bypassing the need for multiple sequence alignments (MSAs). As the model scales, structural information emerges implicitly in its embeddings, enabling accurate and fast structure prediction—up to 60x faster than AlphaFold2 [61]—while maintaining comparable resolution and confidence metrics such as pLDDT. These embeddings can be used with lightweight heads for downstream tasks, making ESM-2 a general-purpose backbone for structure prediction pipelines and allowing generalization across metagenomic and diverse protein families.

**ProtTrans:** This work investigates scaling protein language models using both autoregressive (Transformer-XL [62], XLNet [63]) and auto-encoding models (BERT [64], ALBERT [65], ELECTRA [66], T5 [57]) trained on UniRef and BFD datasets [59, 67]. These PLMs are pretrained in a self-supervised manner, reconstructing corrupted tokens from raw protein sequences where single amino acids act as input tokens. Embeddings—vector representations from the last hidden layer—are extracted and used as exclusive input to downstream models for tasks like secondary structure prediction, subcellular localization, and solubility classification. Auto-encoding models that leverage bidirectional context generally outperform uni-directional autoregressive models, highlighting the importance of capturing full contextual information in protein sequences. In our experiments, we used the ProtBert model for extracting embeddings.

**ESM Cambrian:** A generative PLM family developed alongside ESM-3 [10], designed to learn representations that capture the underlying biology of proteins. It improves upon ESM-2 by scaling up both training data and compute, and is available in 300M, 600M, and 6B parameter versions. Trained using a masked language modeling objective, ESM Cambrian learns biological structure and function from unlabeled protein sequences by capturing patterns shaped by evolution. These internal representations reflect the hidden variables driving amino acid selection, enabling broader generalization than models relying only on labeled structural or functional data.

## Supplementary Material

iqag008_Supplementary_Data

## Data Availability

Data and code implementations can be accessed at https://github.com/alifbinabdulqayyum/DUNE.
