# Supplementary material for: Enhancing protein immunogenicity prediction via uncertainty weighted deep ensemble
Source: Oxf Open Immunol. 2026 Mar 27;7(1):iqag008. doi: 10.1093/oxfimm/iqag008 (PMC13174275; doi:10.1093/oxfimm/iqag008)
Supplement: iqag008_Supplementary_Data [file iqag008_supplementary_data.pdf]

Supplementary Materials

Alif Bin Abdul Qayyum, Amir Hossein Rahmati, Xiaoning Qian
and Byung-Jun Yoon

1 Additional Results

1.1 Experiment 2. Comparative Evaluation of Uncertainty Weighting Approaches

Table 1, 2, 3 and 4 show the comparative results on different weighting approaches with DKL, VBLL, LA and SWAG based BNN to convert the ensemble members into probabilistic models accordingly.

Table 1: Comparative results on different weighting approaches. KLD denotes KL-Divergence weighting and NS denotes Negative-Softmax weighting. All reported results for DUNE methods with each member as a DKL based probabilistic model.

Dataset	Method	Accuracy(↑)	Precision(↑)	Recall(↑)	F1 Score(↑)	AUC ROC(↑)	NLL(↓)
Virus	KLD	0.9332	0.9227	0.9479	0.9351	0.9771	0.2140
	NS(c=5)	0.9395	0.9340	0.9479	0.9409	0.9787	0.2224
	NS(c=25)	0.9332	0.9227	0.9479	0.9351	0.9708	0.2059
	NS(c=100)	0.9320	0.9246	0.9429	0.9337	0.9697	0.2317
	Unbiased	0.9370	0.9294	0.9479	0.9386	0.9755	0.2019
Bacteria	KLD	0.8387	0.8092	0.7069	0.7546	0.8750	0.5049
	NS(c=5)	0.8347	0.7949	0.7126	0.7515	0.8874	0.5513
	NS(c=25)	0.8306	0.7848	0.7126	0.7470	0.8828	0.5795
	NS(c=100)	0.8206	0.7640	0.7069	0.7343	0.8734	0.6380
	Unbiased	0.8327	0.7862	0.7184	0.7508	0.8859	0.5731
Tumor	KLD	0.6795	0.5965	0.5574	0.5763	0.7220	0.6917
	NS(c=5)	0.5705	0.4706	0.7869	0.5890	0.6378	0.6925
	NS(c=25)	0.6474	0.5375	0.7049	0.6099	0.6915	0.6920
	NS(c=100)	0.6538	0.6400	0.2623	0.3721	0.7229	0.6915
	Unbiased	0.6410	0.5472	0.4754	0.5088	0.7053	0.6918
Toxicity^T	KLD	0.9845	0.8661	0.8818	0.8739	0.9899	0.0784
	NS(c=5)	0.9839	0.8584	0.8818	0.8700	0.9897	0.0801
	NS(c=25)	0.9834	0.8636	0.8636	0.8636	0.9709	0.0718
	NS(c=100)	0.9823	0.8611	0.8455	0.8532	0.9356	0.0771
	Unbiased	0.9828	0.8624	0.8545	0.8584	0.9333	0.0756
Toxicity^I	KLD	0.9640	0.4612	0.6645	0.5445	0.9506	0.1160
	NS(c=5)	0.9642	0.4636	0.6711	0.5484	0.9523	0.1168
	NS(c=25)	0.9670	0.4925	0.6513	0.5609	0.9014	0.1134
	NS(c=100)	0.9666	0.4870	0.6184	0.5449	0.8250	0.1281
	Unbiased	0.9668	0.4896	0.6184	0.5465	0.8309	0.1188

1.2 Experiment 3. Comparative Probabilistic Member Architectures

Table 5, 6, 7 and 8 show the results for different probabilistic models with Negative-softmax based weighting approach with $c = 5, 25, 100$ and unbiased weighting approach for DUNE method accordingly.

1.3 Experiment 4. Uncertainty Quantification Evaluation

Table 9, 10, 11 and 12 show the results for different probabilistic models on several uncertainty quantification metrics, along with our proposed Cross-Divergence metric with Negative-softmax based weighting approach with $c = 5, 25, 100$ and unbiased weighting approach.

Table 2: Comparative results on different weighting approaches. KLD denotes KL-Divergence weighting and NS denotes Negative-Softmax weighting. All reported results for DUNE methods with each member as a DVBLL based probabilistic model.

Dataset	Method	Accuracy(↑)	Precision(↑)	Recall(↑)	F1 Score(↑)	AUC ROC(↑)	NLL(↓)
Virus	KLD	0.9307	0.9350	0.9280	0.9315	0.9763	0.2007
	NS(c=5)	0.9332	0.9375	0.9305	0.9340	0.9785	0.1863
	NS(c=25)	0.9320	0.9352	0.9305	0.9328	0.9773	0.1988
	NS(c=100)	0.9320	0.9395	0.9256	0.9325	0.9636	0.3080
	Unbiased	0.9270	0.9345	0.9206	0.9275	0.9692	0.4974
Bacteria	KLD	0.8327	0.7725	0.7414	0.7566	0.8911	0.5628
	NS(c=5)	0.8387	0.7831	0.7471	0.7647	0.8916	0.5052
	NS(c=25)	0.8347	0.7771	0.7414	0.7588	0.8914	0.6053
	NS(c=100)	0.8306	0.7647	0.7471	0.7558	0.8808	0.8285
	Unbiased	0.8306	0.7647	0.7471	0.7558	0.8825	1.6208
Tumor	KLD	0.7628	0.6765	0.7541	0.7132	0.8516	0.5383
	NS(c=5)	0.7692	0.6761	0.7869	0.7273	0.8594	0.4628
	NS(c=25)	0.7885	0.7000	0.8033	0.7481	0.8373	0.5919
	NS(c=100)	0.7885	0.6944	0.8197	0.7519	0.8071	1.0709
	Unbiased	0.7885	0.7000	0.8033	0.7481	0.8223	1.2291
Toxicity^T	KLD	0.9845	0.8596	0.8909	0.8750	0.9909	0.0534
	NS(c=5)	0.9839	0.8522	0.8909	0.8711	0.9904	0.0533
	NS(c=25)	0.9834	0.8509	0.8818	0.8661	0.9909	0.0530
	NS(c=100)	0.9834	0.8571	0.8727	0.8649	0.9915	0.0683
	Unbiased	0.9817	0.8407	0.8636	0.8520	0.9909	0.1365
Toxicity^I	KLD	0.9698	0.5269	0.6447	0.5799	0.9619	0.0860
	NS(c=5)	0.9677	0.5000	0.6645	0.5706	0.9616	0.0883
	NS(c=25)	0.9677	0.5000	0.6447	0.5632	0.9629	0.0910
	NS(c=100)	0.9666	0.4872	0.6250	0.5476	0.9537	0.1208
	Unbiased	0.9664	0.4847	0.6250	0.5460	0.9559	0.2401

2 Case Study

Table 13 shows the list of proteins utilized in the case study.

Table 3: Comparative results on different weighting approaches. KLD denotes KL-Divergence weighting and NS denotes Negative-Softmax weighting. All reported results for DUNE methods with each member as a LA based probabilistic model.

Dataset	Method	Accuracy(↑)	Precision(↑)	Recall(↑)	F1 Score(↑)	AUC ROC(↑)	NLL(↓)
Virus	KLD	0.9232	0.9233	0.9256	0.9244	0.9749	0.2014
	NS(c=5)	0.9207	0.9187	0.9256	0.9221	0.9769	0.2020
	NS(c=25)	0.9194	0.9165	0.9256	0.9210	0.9767	0.1980
	NS(c=100)	0.9257	0.9257	0.9280	0.9269	0.9763	0.1951
	Unbiased	0.9257	0.9236	0.9305	0.9271	0.9651	0.4645
Bacteria	KLD	0.8286	0.7572	0.7529	0.7550	0.8858	0.4784
	NS(c=5)	0.8266	0.7588	0.7414	0.7500	0.8867	0.4265
	NS(c=25)	0.8327	0.7630	0.7586	0.7608	0.8869	0.4388
	NS(c=100)	0.8347	0.7644	0.7644	0.7644	0.8827	0.5092
	Unbiased	0.8347	0.7614	0.7701	0.7657	0.8740	0.9345
Tumor	KLD	0.7500	0.6447	0.8033	0.7153	0.8380	0.5708
	NS(c=5)	0.7436	0.6400	0.7869	0.7059	0.8590	0.4661
	NS(c=25)	0.7372	0.6282	0.8033	0.7050	0.8495	0.4962
	NS(c=100)	0.7436	0.6329	0.8197	0.7143	0.8079	0.7656
	Unbiased	0.7436	0.6329	0.8197	0.7143	0.8053	1.4870
Toxicity^T	KLD	0.9845	0.8661	0.8818	0.8739	0.9904	0.0551
	NS(c=5)	0.9839	0.8649	0.8727	0.8688	0.9901	0.0548
	NS(c=25)	0.9839	0.8649	0.8727	0.8688	0.9901	0.0546
	NS(c=100)	0.9839	0.8649	0.8727	0.8688	0.9901	0.0544
	Unbiased	0.9806	0.8440	0.8364	0.8402	0.9817	0.1475
Toxicity^I	KLD	0.9668	0.4896	0.6184	0.5465	0.9591	0.0961
	NS(c=5)	0.9672	0.4947	0.6184	0.5497	0.9582	0.0962
	NS(c=25)	0.9674	0.4974	0.6250	0.5539	0.9582	0.0960
	NS(c=100)	0.9674	0.4974	0.6316	0.5565	0.9584	0.0954
	Unbiased	0.9664	0.4845	0.6184	0.5434	0.9067	0.2350

Table 4: Comparative results on different weighting approaches. KLD denotes KL-Divergence weighting and NS denotes Negative-Softmax weighting. All reported results for DUNE methods with each member as a SWAG based probabilistic model.

Dataset	Method	Accuracy(↑)	Precision(↑)	Recall(↑)	F1 Score(↑)	AUC ROC(↑)	NLL(↓)
Virus	KLD	0.9345	0.9249	0.9479	0.9363	0.9792	0.1958
	NS(c=5)	0.9320	0.9225	0.9454	0.9338	0.9799	0.1991
	NS(c=25)	0.9207	0.9126	0.9330	0.9227	0.9755	0.1980
	NS(c=100)	0.9207	0.9126	0.9330	0.9227	0.9698	0.2156
	Unbiased	0.9244	0.9153	0.9380	0.9265	0.9765	0.1945
Bacteria	KLD	0.8347	0.7738	0.7471	0.7602	0.8796	0.4200
	NS(c=5)	0.8306	0.7711	0.7356	0.7529	0.8808	0.4086
	NS(c=25)	0.8266	0.7619	0.7356	0.7485	0.8683	0.4541
	NS(c=100)	0.8165	0.7515	0.7126	0.7316	0.8544	0.5571
	Unbiased	0.8286	0.7665	0.7356	0.7507	0.8716	0.4710
Tumor	KLD	0.7500	0.6667	0.7213	0.6929	0.8373	0.4960
	NS(c=5)	0.7628	0.6765	0.7541	0.7132	0.8602	0.4551
	NS(c=25)	0.7821	0.7077	0.7541	0.7302	0.8540	0.4697
	NS(c=100)	0.7628	0.6935	0.7049	0.6992	0.8361	0.5203
	Unbiased	0.7756	0.7031	0.7377	0.7200	0.8547	0.4690
Toxicity^T	KLD	0.9817	0.8235	0.8909	0.8559	0.9932	0.0600
	NS(c=5)	0.9806	0.8049	0.9000	0.8498	0.9924	0.0658
	NS(c=25)	0.9751	0.7519	0.8818	0.8117	0.9899	0.0651
	NS(c=100)	0.9740	0.7442	0.8727	0.8033	0.9888	0.0685
	Unbiased	0.9773	0.7717	0.8909	0.8270	0.9907	0.0611
Toxicity^I	KLD	0.9608	0.4380	0.7434	0.5512	0.9633	0.1099
	NS(c=5)	0.9549	0.3958	0.7500	0.5182	0.9627	0.1180
	NS(c=25)	0.9485	0.3602	0.7632	0.4895	0.9615	0.1193
	NS(c=100)	0.9438	0.3372	0.7632	0.4677	0.9606	0.1274
	Unbiased	0.9511	0.3725	0.7500	0.4978	0.9628	0.1155

Table 5: Comparative results for different probabilistic ensemble member model. Toxicity^T and Toxicity^I denote the test and independent toxicity datasets accordingly. All reported results are for DUNE method with Negative-softmax based weighting strategy with $c = 5$.

Prediction Metric	Model	Virus	Bacteria	Tumor	Toxicity^T	Toxicity^I
Accuracy(↑)	MCD	0.9395	0.8327	0.7436	0.9845	0.9634
	DKL	0.9395	0.8347	0.5705	0.9839	0.9642
	DVBLL	0.9332	0.8387	0.7692	0.9839	0.9677
	LA	0.9207	0.8266	0.7436	0.9839	0.9672
	SWAG	0.9320	0.8306	0.7628	0.9806	0.9549
Precision(↑)	MCD	0.9298	0.8054	0.6400	0.8596	0.4580
	DKL	0.9340	0.7949	0.4706	0.8584	0.4636
	DVBLL	0.9375	0.7831	0.6761	0.8522	0.5000
	LA	0.9187	0.7588	0.6400	0.8649	0.4947
	SWAG	0.9225	0.7711	0.6765	0.8049	0.3958
Recall(↑)	MCD	0.9529	0.6897	0.7869	0.8909	0.7171
	DKL	0.9479	0.7126	0.7869	0.8818	0.6711
	DVBLL	0.9305	0.7471	0.7869	0.8909	0.6645
	LA	0.9256	0.7414	0.7869	0.8727	0.6184
	SWAG	0.9454	0.7356	0.7541	0.9000	0.7500
F1 Score(↑)	MCD	0.9412	0.7430	0.7059	0.8750	0.5590
	DKL	0.9409	0.7515	0.5890	0.8700	0.5484
	DVBLL	0.9340	0.7647	0.7273	0.8711	0.5706
	LA	0.9221	0.7500	0.7059	0.8688	0.5497
	SWAG	0.9338	0.7529	0.7132	0.8498	0.5182
AUC-ROC(↑)	MCD	0.9810	0.8883	0.8475	0.9902	0.9634
	DKL	0.9787	0.8874	0.6378	0.9897	0.9523
	DVBLL	0.9785	0.8916	0.8594	0.9904	0.9616
	LA	0.9769	0.8867	0.8590	0.9901	0.9582
	SWAG	0.9799	0.8808	0.8602	0.9924	0.9627

Table 6: Comparative results for different probabilistic ensemble member model. Toxicity^T and Toxicity^I denote the test and independent toxicity datasets accordingly. All reported results are for DUNE method with Negative-softmax based weighting strategy with $c = 25$.

Prediction Metric	Model	Virus	Bacteria	Tumor	Toxicity^T	Toxicity^I
Accuracy(↑)	MCD	0.9383	0.8327	0.7308	0.9845	0.9638
	DKL	0.9332	0.8306	0.6474	0.9834	0.9670
	DVBLL	0.9320	0.8347	0.7885	0.9834	0.9677
	LA	0.9194	0.8327	0.7372	0.9839	0.9674
	SWAG	0.9207	0.8266	0.7821	0.9751	0.9485
Precision(↑)	MCD	0.9296	0.8054	0.6338	0.8596	0.4619
	DKL	0.9227	0.7848	0.5375	0.8636	0.4925
	DVBLL	0.9352	0.7771	0.7000	0.8509	0.5000
	LA	0.9165	0.7630	0.6282	0.8649	0.4974
	SWAG	0.9126	0.7619	0.7077	0.7519	0.3602
Recall(↑)	MCD	0.9504	0.6897	0.7377	0.8909	0.7171
	DKL	0.9479	0.7126	0.7049	0.8636	0.6513
	DVBLL	0.9305	0.7414	0.8033	0.8818	0.6447
	LA	0.9256	0.7586	0.8033	0.8727	0.6250
	SWAG	0.9330	0.7356	0.7541	0.8818	0.7632
F1 Score(↑)	MCD	0.9399	0.7430	0.6818	0.8750	0.5619
	DKL	0.9351	0.7470	0.6099	0.8636	0.5609
	DVBLL	0.9328	0.7588	0.7481	0.8661	0.5632
	LA	0.9210	0.7608	0.7050	0.8688	0.5539
	SWAG	0.9227	0.7485	0.7302	0.8117	0.4895
AUC-ROC(↑)	MCD	0.9812	0.8881	0.8475	0.9903	0.9631
	DKL	0.9708	0.8828	0.6915	0.9709	0.9014
	DVBLL	0.9773	0.8914	0.8373	0.9909	0.9629
	LA	0.9767	0.8869	0.8495	0.9901	0.9582
	SWAG	0.9755	0.8683	0.8540	0.9899	0.9615

Table 7: Comparative results for different probabilistic ensemble member model. Toxicity^T and Toxicity^I denote the test and independent toxicity datasets accordingly. All reported results are for DUNE method with Negative-softmax based weighting strategy with $c = 100$.

Prediction Metric	Model	Virus	Bacteria	Tumor	Toxicity^T	Toxicity^I
Accuracy(↑)	MCD	0.9370	0.8407	0.7436	0.9839	0.9636
	DKL	0.9320	0.8206	0.6538	0.9823	0.9666
	DVBLL	0.9320	0.8306	0.7885	0.9834	0.9666
	LA	0.9257	0.8347	0.7436	0.9839	0.9674
	SWAG	0.9207	0.8165	0.7628	0.9740	0.9438
Precision(↑)	MCD	0.9253	0.8105	0.6522	0.8462	0.4599
	DKL	0.9246	0.7640	0.6400	0.8611	0.4870
	DVBLL	0.9395	0.7647	0.6944	0.8571	0.4872
	LA	0.9257	0.7644	0.6329	0.8649	0.4974
	SWAG	0.9126	0.7515	0.6935	0.7442	0.3372
Recall(↑)	MCD	0.9529	0.7126	0.7377	0.9000	0.7171
	DKL	0.9429	0.7069	0.2623	0.8455	0.6184
	DVBLL	0.9256	0.7471	0.8197	0.8727	0.6250
	LA	0.9280	0.7644	0.8197	0.8727	0.6316
	SWAG	0.9330	0.7126	0.7049	0.8727	0.7632
F1 Score(↑)	MCD	0.9389	0.7584	0.6923	0.8722	0.5604
	DKL	0.9337	0.7343	0.3721	0.8532	0.5449
	DVBLL	0.9325	0.7558	0.7519	0.8649	0.5476
	LA	0.9269	0.7644	0.7143	0.8688	0.5565
	SWAG	0.9227	0.7316	0.6992	0.8033	0.4677
AUC-ROC(↑)	MCD	0.9807	0.8881	0.8385	0.9908	0.9646
	DKL	0.9697	0.8734	0.7229	0.9356	0.8250
	DVBLL	0.9636	0.8808	0.8071	0.9915	0.9537
	LA	0.9763	0.8827	0.8079	0.9901	0.9584
	SWAG	0.9698	0.8544	0.8361	0.9888	0.9606

Table 8: Comparative results for different probabilistic ensemble member model. Toxicity^T and Toxicity^I denote the test and independent toxicity datasets accordingly. All reported results are for DUNE method with unbiased weighting strategy.

Prediction Metric	Model	Virus	Bacteria	Tumor	Toxicity^T	Toxicity^I
Accuracy(↑)	MCD	0.9282	0.8448	0.7500	0.9839	0.9625
	DKL	0.9370	0.8327	0.6410	0.9828	0.9668
	DVBLL	0.9270	0.8306	0.7885	0.9817	0.9664
	LA	0.9257	0.8347	0.7436	0.9806	0.9664
	SWAG	0.9244	0.8286	0.7756	0.9773	0.9511
Precision(↑)	MCD	0.9159	0.8170	0.6618	0.8462	0.4496
	DKL	0.9294	0.7862	0.5472	0.8624	0.4896
	DVBLL	0.9345	0.7647	0.7000	0.8407	0.4847
	LA	0.9236	0.7614	0.6329	0.8440	0.4845
	SWAG	0.9153	0.7665	0.7031	0.7717	0.3725
Recall(↑)	MCD	0.9454	0.7184	0.7377	0.9000	0.7039
	DKL	0.9479	0.7184	0.4754	0.8545	0.6184
	DVBLL	0.9206	0.7471	0.8033	0.8636	0.6250
	LA	0.9305	0.7701	0.8197	0.8364	0.6184
	SWAG	0.9380	0.7356	0.7377	0.8909	0.7500
F1 Score(↑)	MCD	0.9304	0.7645	0.6977	0.8722	0.5487
	DKL	0.9386	0.7508	0.5088	0.8584	0.5465
	DVBLL	0.9275	0.7558	0.7481	0.8520	0.5460
	LA	0.9271	0.7657	0.7143	0.8402	0.5434
	SWAG	0.9265	0.7507	0.7200	0.8270	0.4978
AUC-ROC(↑)	MCD	0.9711	0.8892	0.8264	0.9931	0.9683
	DKL	0.9755	0.8859	0.7053	0.9333	0.8309
	DVBLL	0.9692	0.8825	0.8223	0.9909	0.9559
	LA	0.9651	0.8740	0.8053	0.9817	0.9067
	SWAG	0.9765	0.8716	0.8547	0.9907	0.9628

Table 9: Uncertainty Quantification Evaluation. Toxicity^T and Toxicity^I denote the test and independent toxicity datasets accordingly. All reported results are for DUNE method with Negative-softmax based weighting strategy with $c = 5$.

UQ Metric	Model	Virus	Bacteria	Tumor	Toxicity^T	Toxicity^I
CDiv(↑)	MCD	6.6719	0.5928	-3.6989	12.4150	9.8370
	DKL	3.5264	0.2320	0.0006	6.6525	6.1032
	DVBLL	5.6971	-4.7628	-0.6719	11.5231	10.3730
	LA	6.1067	0.4271	-1.8109	13.4206	10.8600
	SWAG	4.7570	2.2087	1.6627	8.5277	7.4408
ECE(↓)	MCD	0.0266	0.0703	0.0516	0.0081	0.0359
	DKL	0.0746	0.2224	0.1098	0.0383	0.0582
	DVBLL	0.0108	0.0862	0.0481	0.0068	0.0279
	LA	0.0254	0.0595	0.0437	0.0036	0.0250
	SWAG	0.0480	0.0259	0.0134	0.0214	0.0594
NLL(↓)	MCD	0.1836	0.4800	0.4718	0.0534	0.0979
	DKL	0.2224	0.5513	0.6925	0.0801	0.1168
	DVBLL	0.1863	0.5052	0.4628	0.0533	0.0883
	LA	0.2020	0.4265	0.4661	0.0548	0.0962
	SWAG	0.1991	0.4086	0.4551	0.0658	0.1180
Brier Score(↓)	MCD	0.0516	0.1304	0.1605	0.0138	0.0282
	DKL	0.0557	0.1806	0.2497	0.0144	0.0273
	DVBLL	0.0519	0.1290	0.1549	0.0144	0.0247
	LA	0.0569	0.1255	0.1559	0.0145	0.0262
	SWAG	0.0541	0.1271	0.1519	0.0165	0.0332

Table 10: Uncertainty Quantification Evaluation. Toxicity^T and Toxicity^I denote the test and independent toxicity datasets accordingly. All reported results are for DUNE method with Negative-softmax based weighting strategy with $c = 25$.

UQ Metric	Model	Virus	Bacteria	Tumor	Toxicity^T	Toxicity^I
CDiv(↑)	MCD	6.6719	0.5928	-3.6989	12.4150	9.8370
	DKL	3.5264	0.2320	0.0006	6.6525	6.1032
	DVBLL	5.6971	-4.7628	-0.6719	11.5231	10.3730
	LA	6.1067	0.4271	-1.8109	13.4206	10.8600
	SWAG	4.7570	2.2087	1.6627	8.5277	7.4408
ECE(↓)	MCD	0.0163	0.0777	0.0822	0.0069	0.0326
	DKL	0.0124	0.2436	0.1460	0.0252	0.0397
	DVBLL	0.0207	0.1190	0.1058	0.0034	0.0210
	LA	0.0151	0.0680	0.0833	0.0035	0.0246
	SWAG	0.0026	0.0645	0.0279	0.0154	0.0492
NLL(↓)	MCD	0.1794	0.4880	0.4822	0.0518	0.0969
	DKL	0.2059	0.5795	0.6920	0.0718	0.1134
	DVBLL	0.1988	0.6053	0.5919	0.0530	0.0910
	LA	0.1980	0.4388	0.4962	0.0546	0.0960
	SWAG	0.1980	0.4541	0.4697	0.0651	0.1193
Brier Score(↓)	MCD	0.0502	0.1314	0.1641	0.0134	0.0278
	DKL	0.0545	0.1939	0.2494	0.0140	0.0272
	DVBLL	0.0543	0.1390	0.1825	0.0143	0.0251
	LA	0.0561	0.1284	0.1684	0.0144	0.0261
	SWAG	0.0552	0.1376	0.1565	0.0176	0.0358

Table 11: Uncertainty Quantification Evaluation. Toxicity^T and Toxicity^I denote the test and independent toxicity datasets accordingly. All reported results are for DUNE method with Negative-softmax based weighting strategy with $c = 100$.

UQ Metric	Model	Virus	Bacteria	Tumor	Toxicity^T	Toxicity^I
CDiv(↑)	MCD	6.6719	0.5928	-3.6989	12.4150	9.8370
	DKL	3.5264	0.2320	0.0006	6.6525	6.1032
	DVBLL	5.6971	-4.7628	-0.6719	11.5231	10.3730
	LA	6.1067	0.4271	-1.8109	13.4206	10.8600
	SWAG	4.7570	2.2087	1.6627	8.5277	7.4408
ECE(↓)	MCD	0.0097	0.0910	0.1357	0.0049	0.0262
	DKL	0.0295	0.2795	0.1521	0.0124	0.0227
	DVBLL	0.0471	0.1458	0.1485	0.0090	0.0227
	LA	0.0061	0.0990	0.1802	0.0032	0.0231
	SWAG	0.0205	0.1065	0.0746	0.0125	0.0445
NLL(↓)	MCD	0.1803	0.5245	0.5734	0.0520	0.1041
	DKL	0.2317	0.6380	0.6915	0.0771	0.1281
	DVBLL	0.3080	0.8285	1.0709	0.0683	0.1208
	LA	0.1951	0.5092	0.7656	0.0544	0.0954
	SWAG	0.2156	0.5571	0.5203	0.0685	0.1274
Brier Score(↓)	MCD	0.0501	0.1352	0.1879	0.0133	0.0290
	DKL	0.0609	0.2225	0.2492	0.0156	0.0297
	DVBLL	0.0633	0.1515	0.2026	0.0153	0.0277
	LA	0.0562	0.1385	0.2216	0.0143	0.0259
	SWAG	0.0598	0.1492	0.1636	0.0192	0.0391

Table 12: Uncertainty Quantification Evaluation. Toxicity^T and Toxicity^I denote the test and independent toxicity datasets accordingly. All reported results are for DUNE method with unbiased weighting strategy.

UQ Metric	Model	Virus	Bacteria	Tumor	Toxicity^T	Toxicity^I
CDiv(↑)	MCD	6.6719	0.5928	-3.6989	12.4150	9.8370
	DKL	3.5264	0.2320	0.0006	6.6525	6.1032
	DVBLL	5.6971	-4.7628	-0.6719	11.5231	10.3730
	LA	6.1067	0.4271	-1.8109	13.4206	10.8600
	SWAG	4.7570	2.2087	1.6627	8.5277	7.4408
ECE(↓)	MCD	0.0582	0.1229	0.2172	0.0130	0.0305
	DKL	0.0099	0.2401	0.1397	0.0115	0.0216
	DVBLL	0.0643	0.1617	0.1375	0.0150	0.0290
	LA	0.0620	0.1342	0.2027	0.0179	0.0296
	SWAG	0.0147	0.0658	0.0274	0.0127	0.0448
NLL(↓)	MCD	0.4163	0.8412	1.7150	0.1631	0.2620
	DKL	0.2019	0.5731	0.6918	0.0756	0.1188
	DVBLL	0.4974	1.6208	1.2291	0.1365	0.2401
	LA	0.4645	0.9345	1.4870	0.1475	0.2350
	SWAG	0.1945	0.4710	0.4690	0.0611	0.1155
Brier Score(↓)	MCD	0.0648	0.1472	0.2307	0.0146	0.0336
	DKL	0.0544	0.1908	0.2493	0.0154	0.0280
	DVBLL	0.0680	0.1627	0.1962	0.0166	0.0306
	LA	0.0680	0.1496	0.2380	0.0182	0.0316
	SWAG	0.0539	0.1366	0.1555	0.0169	0.0344

Table 13: List of Proteins used in case study.

NCBI ID	Protein Name
YP 009724390.1	surface glycoprotein
YP 009742617.1	nsp10
YP 009742616.1	nsp9
YP 009742609.1	nsp2
YP 009742612.1	3C-like proteinase
YP 009725312.1	nsp11
YP 009724396.1	ORF8 protein
YP 009742610.1	nsp3
YP 009724397.2	nucleocapsid phosphoprotein
YP 009742614.1	nsp7
YP 009724395.1	ORF7a protein
YP 009725307.1	RNA-dependent RNA polymerase
YP 009725310.1	endoRNAse
YP 009742611.1	nsp4
YP 009724391.1	ORF3a protein
YP 009742608.1	leader protein
YP 009725308.1	helicase
YP 009742613.1	nsp6
YP 009742615.1	nsp8
YP 009725309.1	3'-to-5' exonuclease
YP 009725311.1	2'-O-ribose methyltransferase
YP 009724393.1	membrane glycoprotein
YP 009724392.1	envelope protein
YP 009724394.1	ORF6 protein
YP 009725318.1	ORF7b
YP 009725255.1	ORF10 protein
